# Oncolytic herpes simplex virus propagates tertiary lymphoid structure formation via CXCL10/CXCR3 to boost antitumor immunity

**DOI:** 10.1111/cpr.13740

**Published:** 2024-09-01

**Authors:** Meng‐Jie Zhang, Wen‐Ping Lin, Qing Wang, Shuo Wang, An Song, Yuan‐Yuan Wang, Hao Li, Zhi‐Jun Sun

**Affiliations:** ^1^ State Key Laboratory of Oral & Maxillofacial Reconstruction and Regeneration, Key Laboratory of Oral Biomedicine Ministry of Education, Hubei Key Laboratory of Stomatology, School & Hospital of Stomatology, Frontier Science Center for Immunology and Metabolism, Taikang Center for Life and Medical Sciences Wuhan University Wuhan China; ^2^ Department of Oral Maxillofacial‐Head Neck Oncology, School & Hospital of Stomatology Wuhan University Wuhan China

## Abstract

Inducing tertiary lymphoid structure (TLS) formation can fuel antitumor immunity. It is necessary to create mouse models containing TLS to explore strategies of TLS formation. Oncolytic herpes simplex virus‐1 (oHSV) exhibited intense effects in preclinical and clinical trials. However, the role of oHSV in TLS formation remains to be elucidated. Here, we observed the presence of TLS in 4MOSC1 and MC38 subcutaneous tumour models. Interestingly, oHSV evoked TLS formation, and increased infiltration of B cells and stem‐like TCF1^+^CD8^+^ T cells proliferation. Mechanistically, oHSV increased the expression of TLS‐related chemokines, along with upregulated CXCL10/CXCR3 to facilitate TLS formation. Notably, CXCL10 and CXCR3 were favourable prognostic factors for cancer patients, and closely related with immune cells infiltration. Inhibiting CXCL10/CXCR3 reduced TCF1^+^CD8^+^ T cells and granzyme B expression, and impaired oHSV‐mediated TLS formation. Furthermore, oHSV‐mediated TLS formation revealed superior response and survival rate when combined with αPD‐1 treatment. Collectively, these findings indicate that oHSV recruits stem‐like TCF1^+^CD8^+^ T cells through CXCL10/CXCR3 pathway to propagate TLS formation, and warrants future antitumor immunity development.

## Graphical abstract

Schematic diagram illustrating the induction of TLS using oHSV to boost immunotherapy. oHSV induces TLS formation and activate the immune system. oHSV‐mediated TLS formation upregulates CXCL10/CXCR3 pathway to recruit stem‐like CD8^+^ T cells and enhance immune checkpoint blockade response.

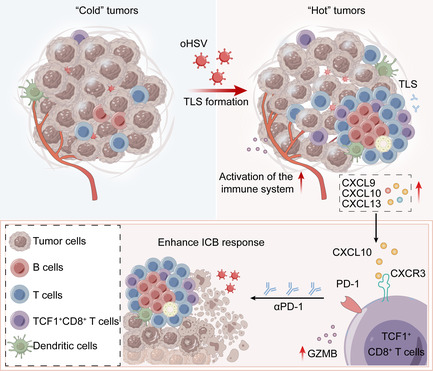

## 1 INTRODUCTION

Immune checkpoint blockade (ICB) therapy, represented by programmed cell death protein 1 pathway (PD‐1/PD‐L1) inhibitors, has marked an unprecedented advancement in the clinical treatment of malignant cancers.^1^, ^2^ Following the Food and Drug Administration's approval of pembrolizumab in 2014, ICB therapy directed against the PD‐1/PD‐L1 signalling pathway has been used for indications spanning a variety of cancers.^3^ Unfortunately, the overall response rate of ICB remains suboptimal, effective within a limited cancer population.^4^ Recent research has emphasised that the efficacy of ICB therapy correlates closely with T cell infiltration in the tumour microenvironment (TME) and the immunogenicity of the tumour.^5^ Therefore, transforming the “cold” tumours defined by little T cell infiltration and poor immunogenicity into the “hot” tumours, marked by substantial T cell infiltration and high immunogenicity is necessary to enhance immunotherapy efficacy.

Inducing the tertiary lymphoid structure (TLS) formation can transform tumours from “cold” to “hot,” and enhance the antitumor immunity in cancer patients.^6^ TLS is organised aggregates of immune cells, predominantly composed of B cells and T cells, formed with the context of chronic inflammation and tumours.^7^ The research highlights that TLS contains progenitor‐exhausted CD8^+^ T cells (CD8^+^ Tpex) displaying high expression of TCF1, which has stem cell‐like properties capable of differentiating into cytotoxic T cells to potentiate ICB therapy.^8^, ^9^ Furthermore, the formation of TLS is associated with the presence of chemokines. Specifically, chemokines, as crucial molecules in the TME, recruit various immune cells to the lymphoid domain, facilitating communication between cells and playing a vital role in supporting TLS formation.^10^, ^11^ To date, TLS has been highlighted in several clinical studies as a meaningful indicator of a better prognosis in many malignancies.^12^, ^13^ However, there are few reports on mouse models with TLS, which limits the exploration of TLS formation. Meanwhile, effective induction strategies for TLS and the specific molecular mechanisms underlying TLS formation are incompletely elucidated.

Oncolytic virotherapy, as the novel immunotherapeutic agents, has shown stable antitumor efficacy in numerous preclinical and clinical trials, because of the ability to release antigens and attract T cells.^14^, ^15^ On the one hand, OVs can specifically kill tumour cells to activate the immunogenic cell death.^16^ On the other hand, OVs release immunogenic molecules to recruit innate immune cells and promote the T cells activation.^17^ Promisingly, studies have demonstrated that OVs have an important effect in inducing TLS formation.^18^ Oncolytic adenovirus with mouse IL‐15 effectively promotes the activation of immune cells, to potently enhance immunotherapy response, and its effectiveness correlates with the presence of TLS.^19^ Therefore, using OVs to induce TLS formation in TME seems to be a promising strategy.^20^ Herpes simplex virus‐1 (oHSV)‐based OVs reprogram the immunosuppressive TME into a more proinflammatory status, which has significant advancements in the field of cancer treatment.^21^ However, it remains unclear the role of oHSV in TLS formation to enhances cancer immunotherapy efficacy.

In this study, we described the role and molecular mechanism of oHSV in inducing TLS formation in tumours, and identified that oHSV therapy recruits stem‐like TCF1^+^CD8^+^ T cells via CXCL10/CXCR3 pathway to propagated TLS formation. We assessed the proportion of TLS formation in 4MOSC1 and MC38 subcutaneous tumour models and investigated the role of oHSV in inducing TLS formation and immune infiltration in the TME. Furthermore, the mechanism of oHSV induces TLS formation and remodels the TME was explored, including the association of CXCL10, and CXCR3 with the cancer patient's prognosis, as well as the impact of blocking CXCL10/CXCR3 pathway on TLS formation. In addition, the role of oHSV‐mediated TLS formation antitumor immunity was investigated. Collectively, this study lays the foundation for future clinical investigations into the formation of TLS induced by OVs, with the aim of boosting antitumor immunity in cancer patients.

## 2 MATERIALS AND METHODS

### 2.1 Production of oncolytic HSV‐1 D34.5/D47‐IpaH9.8

ICP34.5‐ and ICP47‐deficient HSV‐1 (HSV‐1 Δ34.5/Δ47; hereafter designated as oHSV for brevity) was engineered through targeted deletion of the corresponding coding sequences of ICP34.5 and ICP47 within a bacterial artificial chromosome. This manipulation was achieved via a two‐step Red‐mediated recombination approach, as extensively outlined in our prior research.^22^

### 2.2 Cell lines, animal experiments and ethics

Obtained from the Shanghai Model Organisms Center, Inc., the murine colorectal cancer (CRC) cell line MC38 (Cat. NO. NM‐S13) was cultivated in DMEM medium with FBS (10%) of Gibco and penicillin (1%) of HyClone added. The murine head and neck squamous cell carcinoma (HNSCC) cell line 4MOSC1 was kindly gifted by Prof. J. Silvio Gutkind from the University of California San Diego under an MTA (SD‐2017‐202).^23^, ^24^ Cultivation of 4MOSC1 cells occurred in keratinocyte serum‐free medium (K‐SFM) contained penicillin–streptomycin antibiotics (Gibco), mouse epidermal growth factor of 5 ng/mL, and cholera enterotoxin (Sigma‐Aldrich).

In compliance with the regulations under the Experimental Animal Management Regulations approved by the School and Hospital of Stomatology, Wuhan University, all animal experiments obtained formal approval from the Animal Ethics Committee (approval number: S07922080C). Conforming to the ARRIVE (Animal Research: Reporting of In Vivo Experiments) guidelines 2.0, the study was conducted. Accommodated in a specific pathogen‐free (SPF) animal laboratory, C57BL/6 mice of females, aged 6–8 weeks, were subjected to a 12‐h light–dark cycle and had ad libitum access to food and water. Subcutaneous injection of 2 × 10^6^ HNSCC cells 4MOSC1 and 1 × 10^6^ CRC cells MC38 were performed into the right back of C57BL/6 mice, then segregated into randomised groups for the study. Volume (mm^3^) = ½ × length × width^2^ was used to calculate tumour volume. oHSV (5 × 10^5^ pfu per mouse) was administered intratumorally every 4 days. CXCR3 inhibitor of AMG 487 (5 mg/kg; HY‐15319, MedChemExpress) was administered intraperitoneally 1 day before oHSV therapy. Anti‐PD‐1 antibody from BioXcell (BE0146; 200 μg each mouse) was administered intraperitoneally every 3 days. Euthanasia of the mice was conducted via CO_2_ asphyxiation when tumour burden exceeded the maximum acceptable volume of 1500 mm^3^ or at the end of the experiment. Harvested tumour tissues were then subjected to subsequent experimental analysis.

### 2.3 Multiplexed immunohistochemistry (mIHC)

According to the method described previously,^25^ the Opal 7‐Colour Manual IHC Kit was utilised to perform mIHC staining (NEL811001KT; Akoya Biosciences). Primary antibodies, including CD3 (#99940), CD8 (#98941), PD‐1 (#84651), CD19 (#90176), CD31 (#77699) and TCF1 (#2203), were applied to the tissue sections. These above antibodies were purchased from Cell Signalling Technology. Each target was visualised using a spectrum of tyramine signal amplification fluorophores (Opal690, Opal650, Opal620, Opal570, Opal520), followed by nuclear counterstaining with DAPI. Multispectral imaging was conducted utilising the Vectra system (Akoya Biosciences). Data acquisition was performed with the inForm (Akoya Biosciences).

### 2.4 Flow cytometry

Tumours from mice were collected for flow cytometry according to a previously established protocol.^25^ We employed an array of fluorochrome‐conjugated antibodies: Fixable Viability Dye (#65‐0866‐14, eFluor 506, eBioscience), anti‐CD11c (#11‐0114‐82, FITC, eBioscience), anti‐CD4 (#48‐0042‐82, ef450, eBioscience), anti‐PD‐1 (#12‐9981‐81, PE, eBioscience), anti‐CD3 (#11‐0031‐82, FITC, eBioscience), anti‐Foxp3 (#45577382, PC5.5, eBioscience), anti‐CD45 (#103116, APC‐Cy7, BioLegend), anti‐CD19 (#115511, APC, BioLegend), anti‐CD8 (#100734, PC5.5, BioLegend), anti‐MHC‐II (#107630, PC7, BioLegend), anti‐CD25 (#102012, APC, Biolegend), anti‐CD80 (#104708, PE, Biolegend), anti‐CXCR3 (#126505, PE, BioLegend), anti‐CD86 (#105012, APC, Biolegend), anti‐Granzyme B (#372203, APC, Biolegend), TCF1 (#6709S, Alex647, Cell Signalling Technology). Cell subsets were acquired using a flow cytometer of CytoFLEX and the software of CytoExpert. Analysis was carried out using FlowJo software (V.10, TreeStar). Gating strategies are depicted in Figure S9.

### 2.5 Immunohistochemistry (IHC) and immunofluorescence

For IHC staining, tumour tissue sections, initially embedded in paraffin, underwent sequential deparaffinisation and rehydration processes prior to antigen retrieval. Subsequently, these prepared sections were subjected to an overnight incubation with primary antibodies: CD3 (#99940) and CD19 (#90176) from Cell Signalling Technology, CXCL13 (ab199043; Abcam), and CXCL10 (#10937‐1‐AP; Proteintech). Following scanning with a PANNORAMIC MIDI scanner from 3DHISTECH, Budapest, Hungary, the slides underwent quantitative analysis utilising CaseViewer (Version 2.4). About Immunofluorescence staining, primary antibodies, including CD8 (#98941), PD‐1 (#84651) from Cell Signalling Technology, were applied to the tissue sections, followed by fluorophore‐conjugated secondary antibodies. In the end, DAPI was used to counterstain the nuclei. According to the previous method,^26^ the density of TLS was calculated as number/mm^2^ in tumour regions.

### 2.6 Western blot

The Western blot followed the established procedure.^27^ Concisely, following the assessment of protein levels in the acquired samples, electrophoresis was facilitated for SDS‐PAGE. Following protein resolution, they were transferred to polyvinylidene fluoride (PVDF) membranes and subsequently blocked at ambient temperature for 60 minutes. Subsequently, the PVDF membrane was left to incubate in the primary antibody solution, including CXCR3 (#ab288437; Abcam), CXCL10 (#10937‐1‐AP; Proteintech), and β‐actin, overnight under the 4°C. The next day, suitable horseradish peroxidase‐conjugated antibodies (#SA00001‐1; Proteintech; 1:10000 dilution) were applied to the PVDF membranes for a duration of 60 min followed by chemiluminescent detection using an ECL kit from Advansta. Protein visualisation used the Odyssey system from LI‐COR Biosciences, and analysis was conducted with ImageJ software. The experiment was replicated three times.

### 2.7 Quantitative RT‐PCR (qRT‐PCR)

Tumour tissue was extracted to total RNA based on the protocol provided by Axygen. The following steps involved the spectrometer of Shimadzu UV‐2401PC was used to quantify the RNA concentration. Subsequently, Reverse transcription was performed on the total RNA using reverse transcriptase and amplified. The TLS‐related gene expression level was quantified and normalised against β‐actin as a reference gene. Specific materials and methods steps are presented in the supplemental files and Table S1.

### 2.8 ELISA

Tissue lysates were prepared using RIPA buffer (Beyotime) on ice for the quantification of proteins. Subsequent analysis employed commercially available CXCL10 ELISA kit (#RK00056; ABclonal) and CXCR3 ELISA kit (#RK07080; ABclonal).

### 2.9 RNA‐seq and bioinformatics analysis

Specimens were collected from tumour‐bearing mice. The processes of RNA extraction, sequencing libraries and sequencing were contracted to Wuhan IGENEBOOK Biotechnology Co., Ltd (http://www.igenebook.com). The raw data are deposited on GEO (GSE262913). Briefly, total RNA was extracted and all RNA samples were checked for integrity using the Qsep400 instrument. Subsequently, RNA libraries were constructed and sequenced by the Illumina Novaseq 6000 platform. Quantitative expression levels were computed as transcripts per million (TPM) for all annotated genes, and subsequently converted to a logarithmic scale of log_2_ (TPM + 1) for analysis. Differentially expressed genes (DEGs) analysis was conducted utilising the edgeR software within the R programming environment, comparing the Control cohort with the oHSV‐treated cohort. Genes exhibiting significant differential expression were categorised based on a threshold of |log_2_ (fold change)| >0.5 and an adjusted *p* value (*p*.adj) <0.05, and visually represented through heatmaps constructed with the ggpubr, ggplot2, and pheatmap R packages. The log_2_ (fold change) was used as the ranking metric to explore any overrepresented functional term. The enrichment was calculated using the clusterProfiler R package. MSigDB Collections for mice were carried out with the msigdbr R package.

### 2.10 Statistics

All data in the study were examined using GraphPad Prism 8.0 software. All the above data were illustrated as the mean ± standard deviation (SD). A two‐tailed unpaired *t*‐test was utilised to compare two groups, while a one‐way ANOVA with Tukey's multiple comparisons was employed for comparing multiple groups. To determine the connection in the two groups, the Spearman correlation test was employed. Univariate survival analysis was performed using the Kaplan–Meier curve and determined using a log‐rank (Mantel‐Cox) test for survival. The *p*‐value less than 0.05 was described as statistically significant (*p < 0.05; **p < 0.01; ***p < 0.001).

## 3 RESULTS

### 3.1 The presence of TLS in 4MOSC1 and MC38 tumour‐bearing mouse model

**FIGURE 1:**
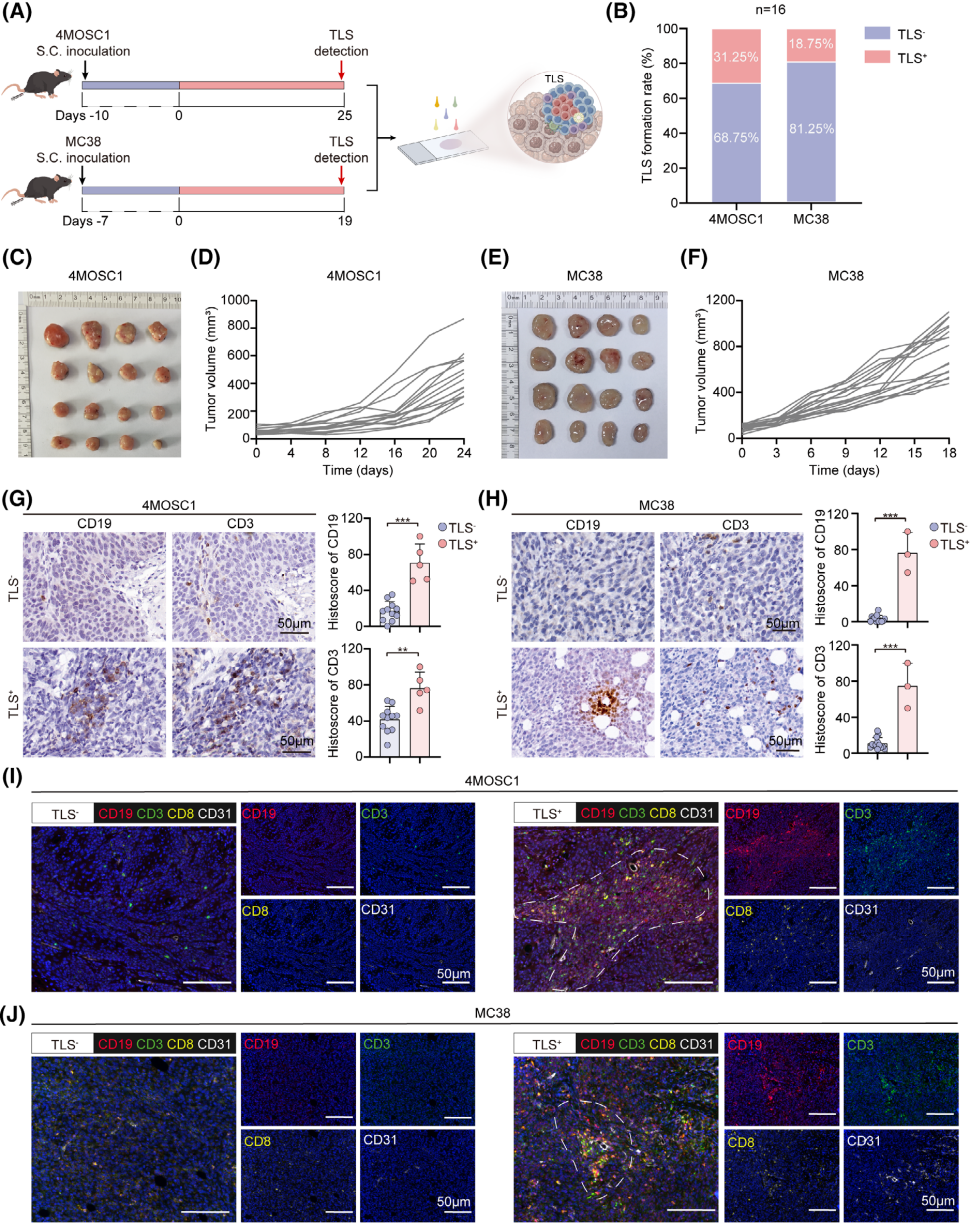
Tertiary lymphoid structure (TLS) is present in the 4MOSC1 and MC38 subcutaneous inoculation tumour model. (A) Experimental schedule of the construction of 4MOSC1 and MC38 tumour‐bearing mice and the strategy for detecting TLS in tumours. (B) The proportion of TLS^+^ and TLS^−^ in 4MOSC1 and MC38 tumour‐bearing mice. (C–F) The tumour images and tumour growth curve of 4MOSC1 and MC38 tumour‐bearing mice (*n* = 16, each group). (G) Representative immunohistochemistry images and quantification of immunohistochemistry of CD19 and CD3 in TLS^−^ and TLS^+^ 4MOSC1 tumour‐bearing mice. (H) Representative immunohistochemistry images and quantification of immunohistochemistry of CD19 and CD3 in TLS^−^ and TLS^+^ MC38 tumour‐bearing mice. (I, J) Representative multiplexed immunohistochemistry (mIHC) images of CD19 (red), CD3 (green), CD8 (yellow), and CD31 (white) in TLS^−^ and TLS^+^ group of 4MOSC1 and MC38 tumour‐bearing mice. Statistical analysis is used by a two‐tailed unpaired *t‐*test. The above data are presented as mean ± SD (**p < 0.01 and ***p < 0.001). Scale bar, 50 μm.

To examine whether the presence of TLS and its proportion in a mouse model, the murine HNSCC cell line 4MOSC1 and murine CRC cell line MC38 were established to verify the TLS by immunohistochemistry (IHC) and multiplexed immunohistochemistry (mIHC) (Figure 1A). Tumour growth curves, tumour images and body weight were examined in 4MOSC1 and MC38 tumour‐bearing mice (Figure 1C–F and Figure S1A–D). The mouse tumours were used for subsequent experiments at 25 and 19 days, respectively.

Considering the characteristics of TLS, the central region of B cells is encircled by T cells, and IHC staining of B cells with CD19 and T cells with CD3 in the 4MOSC1 and MC38 tumour tissues was performed. The findings indicated that 11 of the 16 mice with 4MOSC1 tumours were TLS negative (TLS^−^) and 5 were TLS positive (TLS^+^), with a TLS^+^ proportion of 31.25% (Figure 1B). Meanwhile, 13 of the 16 mice with MC38 tumours were TLS negative (TLS^−^) and 3 were TLS positive (TLS^+^), with a TLS^+^ proportion of 18.75% (Figure 1B). The immunohistochemical expression of CD19 and CD3 in the TLS^+^ group was significantly higher compared to the TLS^−^ group in both tumour models (Figure 1G,H). Similarly, infiltration and aggregation of B cells with CD19 and T cells with CD3 and CD8 were investigated by mIHC images in the group of 4MOSC1 and MC38 tumour tissues with TLS^+^ (Figure 1I,J). The above results suggested TLS exited in 4MOSC1 and MC38 mouse models, which provides dramatically promise for finding TLS induction strategies.

### 3.2 oHSV induces TLS formation and increases stem‐like TCF1 ^+^ CD8 ^+^ T cells

**FIGURE 2:**
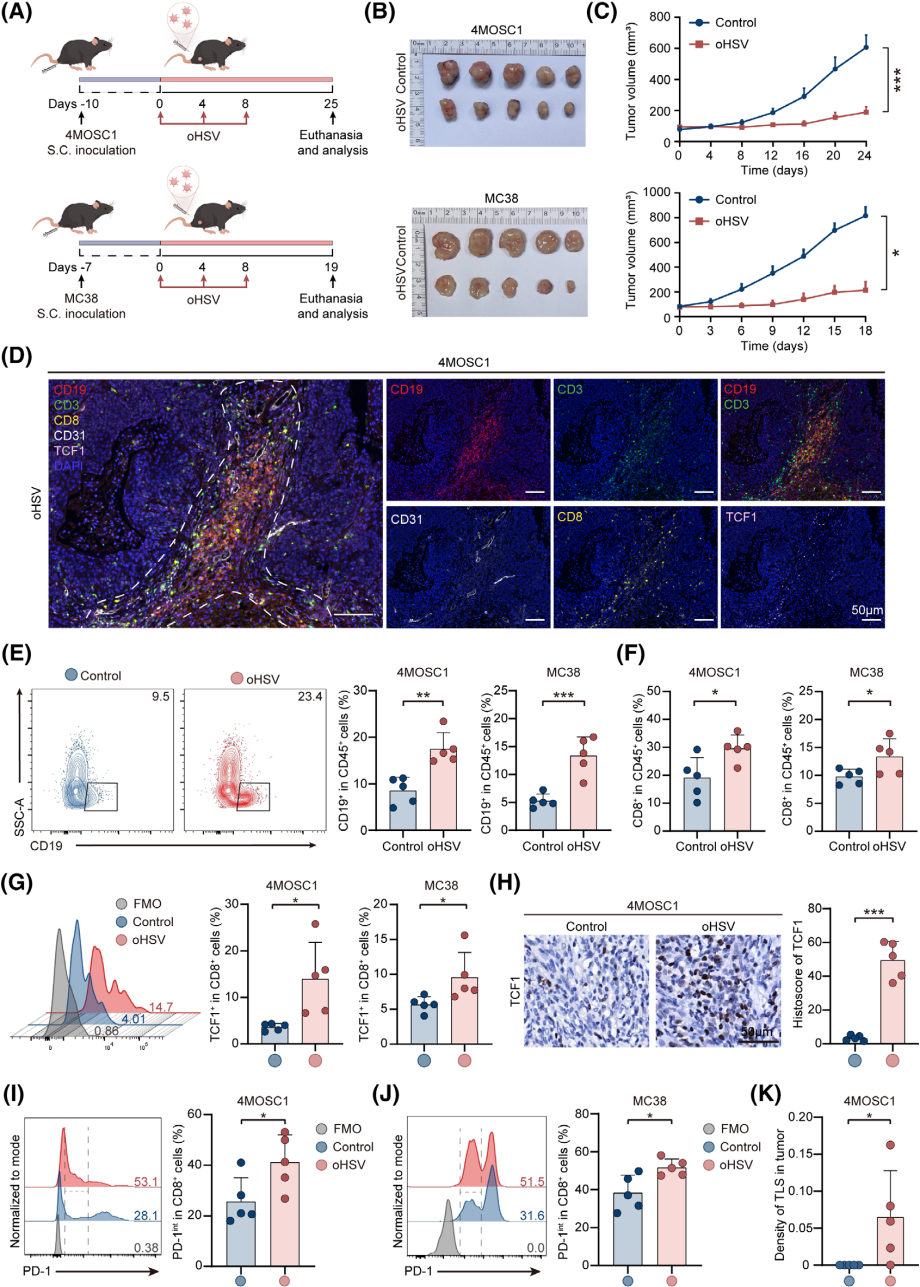
OHSV induces TLS formation in 4MOSC1 and MC38 tumour mouse models. (A) Experimental schedule for 4MOSC1 and MC38 subcutaneous inoculation and treatment with oHSV. (B) The tumour images of the control group and oHSV group. (C) Recording growth curve of tumour volume in control and oHSV groups of 4MOSC1 and MC38 tumour‐bearing mice (*n* = 5, each group). (D) Representative mIHC images of CD19 (red), CD3 (green), CD8 (yellow), CD31 (white), and TCF1 (pink) in 4MOSC1‐bearing mice after oHSV therapy. (E) Analysing images using flow cytometry and quantifying CD19^+^ in CD45^+^ cells of 4MOSC1 tumours and MC38 tumours. (F) Quantifying CD8^+^ cells within CD45^+^ T cells in tumours using flow cytometry. (G) Analysing images using flow cytometry and quantifying TCF1^+^ cells in CD8^+^ T cells in tumours. (H) Representative immunohistochemistry images and quantification of immunohistochemistry of TCF1 in 4MOSC1 tumour‐bearing mice. (I, J) Analysing images by flow cytometry and quantifying PD‐1^int^ in CD8^+^ cells in tumours. (K) Quantifying the density of TLS in control and oHSV groups. Statistical analysis is used by a two‐tailed unpaired *t‐*test. The above data are presented as mean ± SD (*p < 0.05, **p < 0.01 and ***p < 0.001). Scale bar, 50 μm.

To identify whether oHSV can induce TLS formation and the role of oHSV in this process, we used 4MOSC1 and MC38 mouse model treatment with oHSV to elucidate subsequent detection (Figure 2A). In terms of tumour therapy, the tumour images and growth curve showed that the oHSV therapy group effectively suppressed tumour growth of both tumour models (Figure 2B,C and Figure S2A–F). Interestingly, mIHC images revealed a higher infiltration and structurally more organised aggregation of B cells with CD19 and T cells with CD3 and CD8 in the group of 4MOSC1 and MC38 tumour tissues with TLS^+^ (Figure 2D, Figures S2G and S3). The levels of CD31 and TCF1 exhibited a substantial increase in the TLS area after oHSV therapy (Figure 2D). In addition, the density of TLS increased in the oHSV group (Figure 2K and Figure S4A).

Subsequently, flow cytometry was used to confirm that oHSV facilitates TLS formation and remodels the TME. The proportion of CD19^+^ B cells in 4MOSC1 tumours and MC38 tumours increased significantly in the oHSV group (Figure 2E). CD8^+^ T cells infiltration of 4MOSC1 and MC38 tumours was in line with the mIHC images (Figure 2F). Dendritic cells (DCs), as the major antigen‐presenting cells, can present antigen signals to activate T cell proliferation,^28^ our results showed that mature DCs increased in the oHSV group of 4MOSC1 tumours and MC38 tumours (Figure S4D,E). A highly heterogeneous group of exhausted CD8^+^ T cells includes “terminally exhausted” (CD8^+^ Tex) and CD8^+^ Tpex. It has been indicated in research that CD8^+^ Tpex cells with low PD‐1 and high TCF1 expression can self‐renew and replicate, which is essential for TLS formation.^29^, ^30^ Focusing on the impact of oHSV therapy on T cell status, we further examined CD8^+^ Tpex cells. The results showed that TCF1^+^CD8^+^ T cells significantly increased in 4MOSC1 and MC38 tumour cells (Figure 2G,H and Figure S4B,C). We also noted a rise in PD‐1^int^ CD8^+^ T cells in the oHSV‐treated group in comparison to the control group, suggesting oHSV therapy increases CD8^+^ Tpex in the TME (Figure 2I,J). Moreover, in the oHSV group, the proportion of regulatory T cells (Treg), characterised by immunosuppressive effects, decreased compared to the control group (Figure S4F,G **)**. Therefore, we considered that oHSV induces TLS formation by improving the TME, which results in more aggregation of B and T cells. This impact might play a vital effect in enhancing the immunotherapy efficacy of cancers. More importantly, the increase of stem‐like TCF1^+^CD8^+^ Tpex cells and the decrease of Treg are related to the formation of TLS.

### 3.3 oHSV releases TLS‐related 12‐chemokines signature and upregulates CXCL10/CXCR3 pathway

**FIGURE 3:**
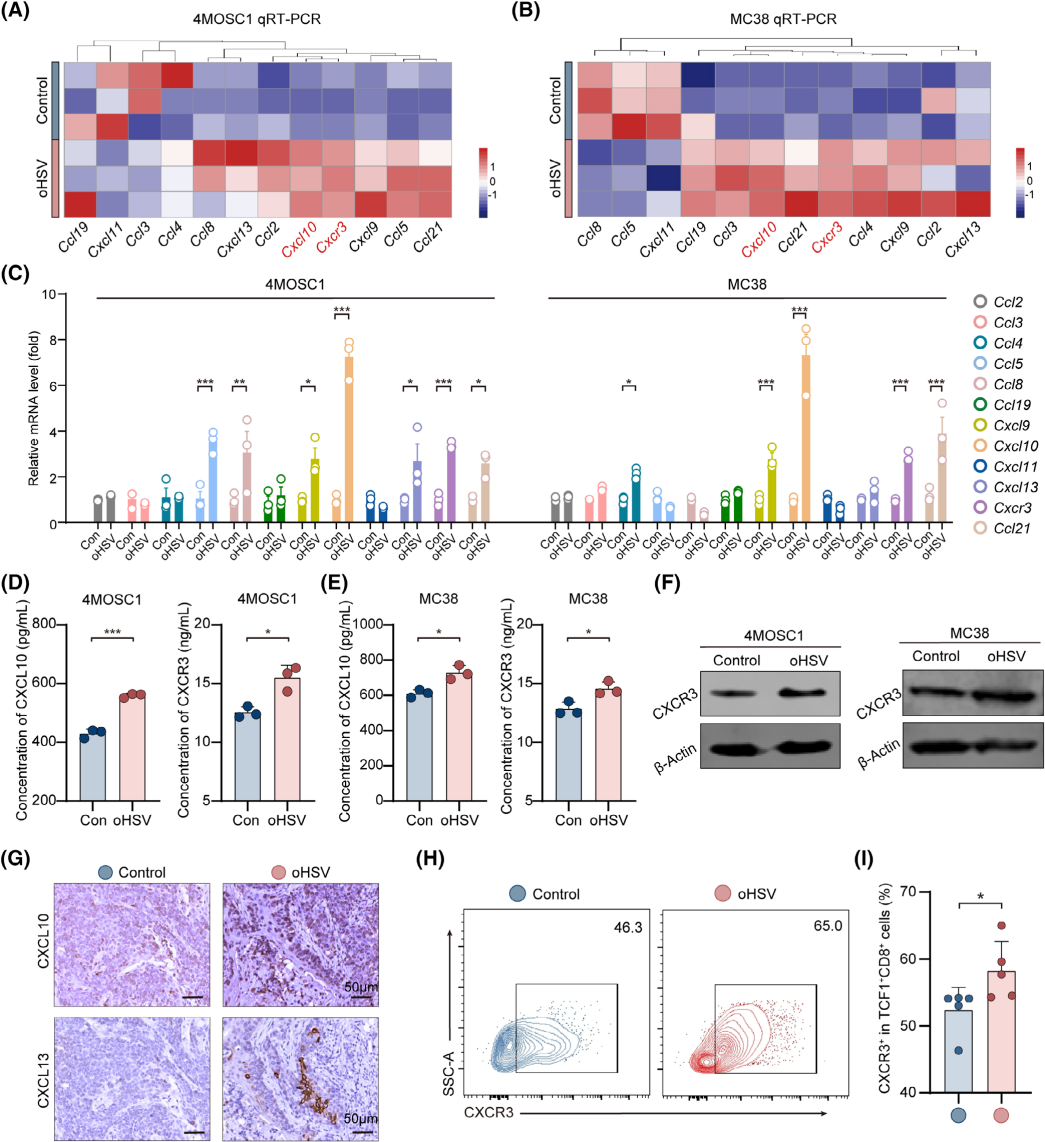
OHSV releases TLS‐related chemokines and upregulates the CXCL10/CXCR3 signalling pathway. (A–C) The heatmap and quantification of the relative RNA expression of chemokines in control and oHSV group (*n* = 3, each group). (D, E) Assessing the CXCL10 and CXCR3 expression at protein level in tumours of the control and oHSV groups measured by ELISA. (F) Assessing the CXCR3 expression at protein level in tumours of the control and oHSV measured by Western Blot. (G) The images using immunohistochemistry of CXCL10 and CXCL13 in 4MOSC1‐bearing mice. (H, I) Analysing images using flow cytometry and quantifying CXCR3^+^ in TCF1^+^CD8^+^ cells. Statistical analysis is used by two‐tailed unpaired *t* test. The above data are presented as mean ± SD (*p < 0.05, **p < 0.01 and ***p < 0.001). Scale bar, 50 μm.

Recent reports have confirmed that 12‐chemokines signature, as TLS‐related genes, play important roles in identification of TLS and TLS formation.^31^ The OVs exhibits potent pro‐inflammatory activity, releasing antigens which generate inflammatory and chemokines.^32^ Therefore, the expression of TLS‐related 12‐chemokines gene signatures was examined. The relative RNA expression demonstrated that *Ccl5*, *Ccl8*, *Cxcl9*, *Cxcl10*, *Cxcl13* and *Ccl21* were significantly higher in the 4MOSC1 tumours following oHSV therapy (Figure 3A,C). In addition, the oHSV treatment resulted in higher expressions of *Ccl4*, *Cxcl9*, *Ccl21* and *Cxcl10* in the MC38 tumours (Figure 3B,C). Notably, the *Cxcl10* gene revealed the most significant change in both tumour models. The results from ELISA demonstrated an upregulation of CXCL10 expression in both 4MOSC1 and MC38 tumours (Figure 3D,E). IHC images showed the CXCL10 and CXCL13 expression was significantly elevated in the oHSV group (Figure 3G).

Mechanistically, focusing on investigating the molecular mechanism of chemokines in the formation of TLS, the CXCL10/CXCR3 pathway was explored. We further investigated the expression of CXCR3, as the CXCL10 receptor, which is consistent with the above results in the oHSV group (Figure 3D–F). In addition, CXCR3 expression showed increase in TCF1^+^CD8^+^ T cells of the oHSV group (Figure 3H,I), consistent with previously reported about the roles of CXCR3 in tumour immunotherapy.^33^ Overall, the above results suggested that oHSV‐mediated TLS formation increases the release of TLS‐related chemokines signatures, especially the *Cxcl10* gene, and upregulates the CXCR3 in stem‐like CD8^+^ T cells.

### 3.4 CXCL10 and CXCR3 expression are significantly correlated with a favourable prognosis and CD8 ^+^ T cells

**FIGURE 4:**
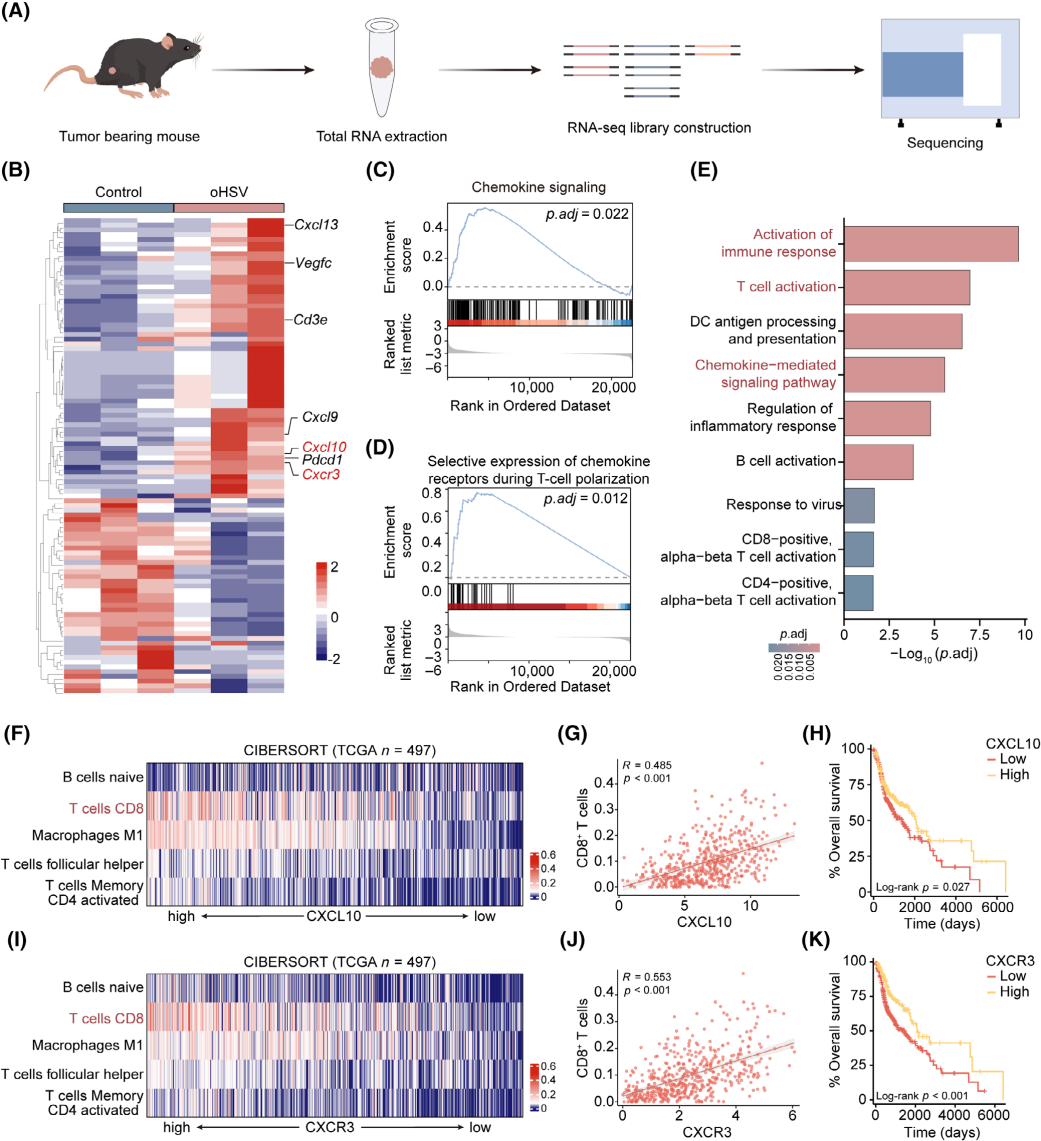
The expression of CXCL10 and CXCR3 is significantly correlated with the good prognosis and immune cell infiltration. (A) Experimental procedures for RNA sequencing of tumour tissues in tumour‐bearing mice. (B) Heatmap illustrating the gene expression contrast between tumours in the control and oHSV therapy groups (*n* = 3, each group). (C) Performing Gene Set Enrichment Analysis (GSEA) to assess the chemokine signalling pathway in tumours from the control and oHSV group. (D) GSEA for expression of chemokine receptors during T‐cell polarisation for tumours in the control and oHSV group. (E) Evaluation of GO and Kyoto Encyclopedia of Genes and Genomes (KEGG) enrichment. (F) The dispersion of B cells naive, T cells CD8, macrophage M1, T cells follicular helper and T cells memory CD4 activated into the tumour site and CXCL10 expression (*n* = 497). (G) The relationship between CXCL10 gene expression and CD8^+^ T cells. (H) Assessing the TCGA‐HNSCC cohort for survival based on CXCL10 expression levels. (I) The dispersion of B cells naive, T cells CD8, macrophage M1, T cells follicular helper and T cells memory CD4 activated into the tumour site and CXCR3 expression (*n* = 497). (J) The relationship between CXCR3 gene expression and CD8^+^ T cells. (K) Survival analysis of TCGA‐HNSCC cohort based on CXCR3 expression level. The correlation was tested by the Spearman correlation test.

To further validate the expression of chemokines and immune‐related markers, we performed the RNA sequencing of 4MOSC1 tumour tissues (Figure 4A). The expression of *Cxcl10* and *Cxcr3* analysed by RNA‐seq was upregulated in TLS^+^ group induced by oHSV therapy (Figure 4B), consistent with above results. In addition, chemokines of *Cxcl9* and *Cxcl13*, *Cd3e*, *Vegfc*, and *Pdcd1* were also significantly increased in the oHSV group (Figure 4B). Meanwhile, chemokine signalling pathway and expression of chemokine receptors during T cell polarisation pathway were significantly enriched in the oHSV group according to Gene set enrichment analysis (GSEA) (Figure 4C,D and Table S2). Meanwhile, oHSV group was significantly enriched in activation of immune response and T cell activation (Figure 4E, Tables S3 and S4, Figure S5A,B). Moreover, the oHSV group also showed a substantial enrichment in B cell activation (Figure 4E and Figure S5B).

The relationship between CXCL10 and CXCR3 expression and immune cell infiltration was assessed using CIBERSORT with the built‐in LM22 signature matrix (LM22). The results suggested that CXCL10 and CXCR3 were widely expressed in TME, and expression was associated with the infiltration of several immune cells (Figure 4F,I). A significant connection was observed between the presence of CD8^+^ T cells and the levels of CXCL10 and CXCR3 expression (Figure 4G,J). Moreover, M1 macrophage and T cells follicular helper are also significant with CXCL10 and CXCR3 expression. The infiltration of B cells demonstrated a strong correlation with CXCR3 expression, but not with CXCL10 expression (Figure S5C–F). In addition, a notable connection was identified between the CXCL10 and CXCR3 genes and the B cell and T cell marker genes, including CD8A and CD19 (Figure S5G–J). Elevated CXCR3 and CXCL10 expression exhibited a significant correlation with improved overall survival in the TCGA‐HNSCC database (Figure 4H,K). According to our sequencing data and TCGA database, CXCL10 and CXCR3 gene expression was strongly associated with favourable prognosis and the abundance of tumour‐infiltrating CD8^+^ T cell populations.

### 3.5 Inhibiting CXCL10/CXCR3 pathway reduces TCF1 ^+^ CD8 ^+^ T cells and affects TLS formation

**FIGURE 5:**
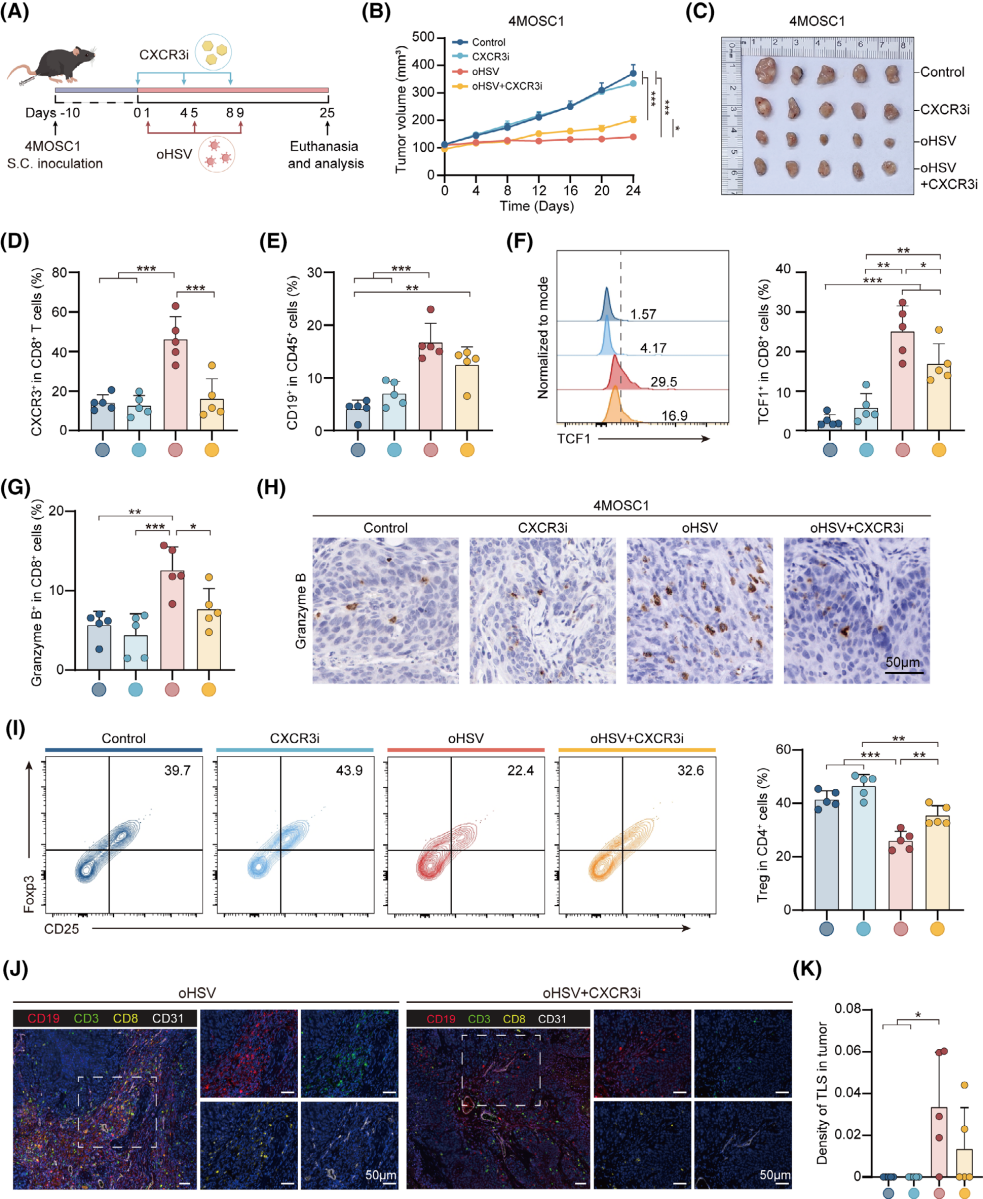
Inhibition of CXCL10/CXCR3 reduces CD8^+^TCF1^+^ T cell aggregation and dampens the TLS formation induced by oHSV therapy. (A) Experimental Schematic for 4MOSC1 subcutaneous tumour inoculation and treatment with oHSV and CXCR3 inhibitory (CXCR3i). (B) Recording the 4MOSC1 tumour growth curve in control and other treatment groups (*n* = 5, each group). (C) Images of tumours in 4MOSC1 tumour‐bearing mice with control, CXCR3i, oHSV, and CXCR3i + oHSV groups. Quantifying CXCR3^+^ in CD8^+^ T cells (D) and CD19^+^ in CD45^+^ cells (E) in tumours using flow cytometry. (F) Analysing images using flow cytometry and quantifying TCF1^+^ in CD8^+^ cells in tumours. (G) Quantifying granzyme B^+^ cells within CD8^+^ cells in tumours using flow cytometry. (H) The images by immunohistochemistry of granzyme B in tumours. (I) Analysing images by flow cytometry and quantifying CD25^+^Foxp3^+^ cells in CD4^+^ cells in tumours. (J) Representative mIHC images of CD19 (red), CD3 (green), CD8 (yellow), CD31 (white) in 4MOSC1‐bearing mice after oHSV or combined therapy. (K) Quantifying the density of TLS in different groups. Statistical analysis is used by one‐way ANOVA with Tukey's multiple comparisons. The above data are presented as mean ± SD (*p < 0.05, **p < 0.01 and ***p < 0.001). Scale bar, 50 μm.

Recent researches have shown that blocking CXCR3 could blocks the CXCL10/CXCR3 axis and inhibit the recruitment of CD8^+^ T cells.^34^, ^35^ To determine whether the CXCL10/CXCR3 would affect TLS formation in the TME and the effect of immune cells, including CD8^+^ T cells status, AMG 487, as the CXCR3 inhibitor (CXCR3i) in preclinical studies, was combined with oHSV therapy in the 4MOSC1 mouse model (Figure 5A). The tumour treatment effect of the oHSV group and combination groups was better than control group and CXCR3i group (Figure 5B). However, we observed that CXCR3i damages the antitumor effects of oHSV therapy (Figure 5B,C) and inhibits CXCR3 expression in CD8^+^ T cells of tumours (Figure 5D and Figure S6A).

Flow cytometry showed that a substantially higher proportion of B cells in oHSV group in comparison to both the control group and CXCR3i group (Figure 5E). Although the proportion of B cells in combination therapy group was not statistically significant compared with the oHSV group, showing a downward trend (Figure 5E and Figure S6B). The combination therapy group showed no statistically significant variance in CD8^+^ T cells compared to the oHSV group, showing a decreasing trend (Figure S6C,D). Notably, the combination therapy group displayed a decline in the expression of stem‐like TCF1^+^CD8^+^ T cells in comparison to the oHSV group (Figure 5F). We further explored that the numbers of granzyme B in CD8^+^ T cells, a maker of T cell activation, which showed an elevation in the oHSV group in contrast to the control and CXCR3i groups (Figure 5G). However, the proportion of granzyme B in tumour cells was impaired in the combination therapy group compared to the oHSV group (Figure 5G and Figure S6E). The IHC image results were also consistent with the above findings (Figure 5H). In contrast to the oHSV group, the combination therapy group exhibited a greater proportion of Treg cells (Figure 5I), suggesting that the immunosuppressive effect of T cells was impaired. mIHC images showed that combination therapy group impaired TLS formation compared with the oHSV group (Figure 5J and Figure S7A,B). TLS density in oHSV was significantly higher than that in control and CXCR3i groups (Figure 5K). In conclusion, above results highlight that CXCR3i impairs oHSV‐mediated TLS formation and reduces stem‐like CD8^+^ T cells and its activation.

### 3.6 Inducing TLS formation by oHSV therapy amplifies the effectiveness of αPD‐1 immunotherapy

**FIGURE 6:**
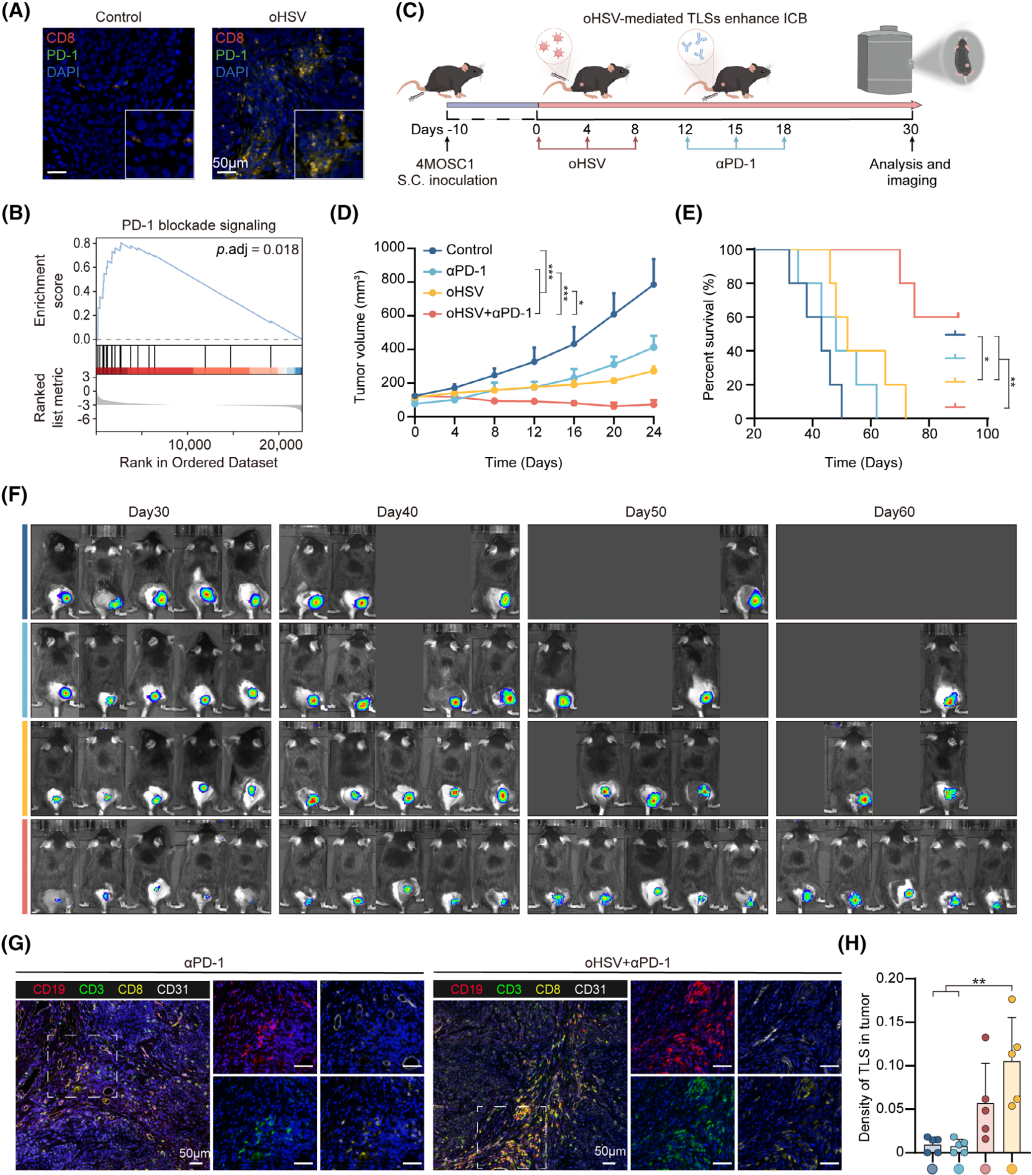
OHSV therapy induces TLS formation to augment the αPD‐1‐mediated antitumor immunity. (A) The images by immunofluorescence of CD8 (red), PD‐1 (green) in 4MOSC1 tumour‐bearing mice after oHSV therapy. (B) GSEA for PD‐1 blockades for tumours in control and oHSV groups. (C) Experimental Schematic for 4MOSC1 subcutaneous tumour inoculation and treatment with oHSV and anti‐PD‐1 antibody (αPD‐1). (D) Recording the 4MOSC1 tumour growth curve in control and other treatment groups (*n* = 5, each group). (E) Analysis of survival among mice in various groups (*n* = 5, each group). (F) Bioluminescence images to track the tumour growth of different groups in vivo on days 30, 40, 50 and 60. (G) Representative mIHC images of CD19 (red), CD3 (green), CD8 (yellow) and CD31 (white) in 4MOSC1‐bearing mice with different treatment. (H) Quantifying the density of TLS in different groups. Statistical analysis is used by one‐way ANOVA with Tukey's multiple comparisons. The above data are presented as mean ± SD (*p < 0.05, **p < 0.01 and ***p < 0.001). Scale bar, 50 μm.

The simultaneous expression of PD‐1 and CD8 was increased in the oHSV group by immunofluorescence images (Figure 6A and Figure S8A) and GSEA showed that the PD‐1 blockade signalling pathway was upregulated in the oHSV group (Figure 6B). These findings give the promising probability for improving the PD‐1 mediated antitumor immunity by oHSV therapy. To elucidated the role of oHSV‐mediated TLS formation in immunotherapy efficiency, we established the oHSV‐mediated TLS^+^ 4MOSC1 tumour‐bearing mouse model (oHSV group) treated with αPD‐1 antibody (Figure 6C). The tumour curve showed that oHSV + αPD‐1 group had a better tumour treatment effect than oHSV group (Figure 6D). Similarly, the oHSV + αPD‐1 group had a better treatment outcome than αPD‐1 group (Figure 6D). Furthermore, based on survival curve and tumour imaging, we observed that the oHSV group combined with αPD‐1 had a longer survival time in comparison to the control, αPD‐1 and oHSV groups (Figure 6E,F). mIHC images revealed a higher infiltration of CD19^+^ B and CD3^+^CD8^+^ T cells in the oHSV combined with αPD‐1 group (Figure 6G). Meanwhile, TLS density of oHSV+PD‐1 group was significantly higher in tumours than other groups (Figure 6H). Structurally, the oHSV combined with αPD‐1 had a more regular spatial aggregation of B and T cells than the αPD‐1 group (Figure 6G and Figure S8B). Consequently, we proposed that oHSV combined with αPD‐1 induced TLS formation, enhancing the efficacy of PD‐1‐mediated cancer immunotherapy.

## 4 DISCUSSION

In this study, oHSV induces TLS formation to enhance the cancer immune response rate and improve the prognosis. 4MOSC1 and MC38 subcutaneous inoculation tumour models were employed to assess the proportion of TLS formation and explore strategies for TLS induction. Our results showed that TLS appeared in 5 out of 16 tumours (31.25%) in the HNSCC mouse model and 3 out of 16 (18.75%) tumours in the CRC mouse model. Consistent with previous reports,^36^, ^37^ the TLS‐positive tumours displayed an increased presence of infiltrating B and T cells compared to the TLS^−^ group. Interestingly, we found that the tumour growth rate of the 4MOSC1 subcutaneous mouse model was relatively slow, suggesting a higher possibility of TLS formation. Although TLS exists in mice with HNSCC and CRC mouse models, the proportion of immune cell infiltration was relatively low. These findings provide opportunities for exploring effective strategies to induce TLS formation through increasing immune cell infiltration.

To further investigate the strategy of inducing TLS formation, 4MOSC1 and MC38 subcutaneous inoculation tumour model were used to detect TLS and the immune status in TME after OVs therapy. To date, four OVs have received global approval for the clinical cancer treatment, including HSV‐1 and adenovirus.^38^ In recent years, there are increasing evidence that OVs have promising therapeutic effects in clinical trials across various human cancers, including CRC and HNSCC.^39^, ^40^ OVs have shown promising potential in releasing antigens to attract immune cells for cancer immunotherapy.^38^ Our results also showed that oHSV significantly inhibited tumour growth and mediated TLS formation in HNSCC and CRC mouse model. More importantly, as a transcription factor within the classical Wnt signalling pathway, TCF‐1 plays a crucial role in the self‐renewal of stem‐like CD8^+^ T cells in reaction to viral or tumour antigens, and augments the response to ICB. Moreover, TCF1^+^CD8^+^ T cells are characterised by self‐renewal, showing a significant correlation with TLS formation.^41^ Consistent with the above evidence, we confirmed that oHSV therapy increased TCF1 expression and aggregation in the TLS region. There are different effects of cancer immunotherapy according to the state of the T cells.^42^ Treg is highly infiltrated in TLS with the potential for tumour escape and is associated with poor prognosis.^43^ We also detected a significant decrease of Tregs in oHSV treatment group.

Previous studies have shown that chemokines are important participants in TLS, recruiting immune cells to the TME.^10^ OVs release a variety of chemokines to recruit and activate antitumor B and CD8^+^ T cells.^15^ Mechanically, we discovered a notable increase in multiple chemokines in the oHSV group through qRT‐PCR and ELISA, with CXCL10 being particularly noteworthy. CXCR3, as a chemokine receptor expressed in T cells, is related to the activation and function of T cells.^44^, ^45^ Our results suggested that CXCR3 expressed on CD8^+^ T cells was upregulated in the oHSV therapy group. Enhanced intratumorally infiltration of CD8^+^ T cells is related to an improved prognosis across various human cancers.^46^ In this study, based on the HNSCC‐TCGA database, we observed that CXCL10 and CXCR3, as markers of favourable prognosis, had a robust relation with CD8^+^ T cells. In addition, cytotoxic T cells (CTL) release cytotoxic molecules, such as granzyme B, to exert functions of killing tumour cells.^47^ We further confirm that the inhibition of the CXCL10/CXCR3 pathway could reduce stem‐like TCF1^+^CD8^+^ T cells and granzyme B expression to impaired oHSV‐mediated TLS formation. However, TLS formation is a complex process, and the relationship between TLS and multiple chemokine networks is still unclear. Further experiments focusing on multiple chemokine networks are needed.^48^ Taken together, above results suggested that oHSV could upregulate CXCL10/CXCR3 pathway to increase TCF1^+^CD8^+^ T cells infiltration to mediate TLS formation.

Recently, TLS formation has been described as a favourable prognostic marker for malignancies.^49^ In clinical studies of non‐small cell lung cancer (NSCLC), patients whose tumours are enriched with TLSs show a better response to neoadjuvant anti‐PD1 therapy.^50^ Our results also indicated that the PD‐1 signalling pathway was enriched in the oHSV group, which provided a promise for the combination of anti‐PD‐1 antibodies. Additionally, oHSV‐mediated TLS exhibited a higher response rate to αPD‐1 and longer survival time.

In this study, we mainly investigated the proportion of TLS in the mouse models and explored the potential therapeutic strategy and molecular mechanisms of inducing TLS to improve the immunotherapy response. Collectively, these results revealed that oHSV is an effective strategy for activating immunity to evoke TLS formation through the CXCL10/CXCR3 pathway, which can enhance immunotherapy efficacy. Considering these promising findings, the oHSV‐mediated TLS formation is expected to improve the prognosis of cancer patients and fuel antitumor immunity.

## AUTHOR CONTRIBUTIONS

**Meng‐Jie Zhang** and **Wen‐Ping Lin**: contributed to the design of research studies, conducted experiments, data acquisition, interpretation, and analysis, drafted and revised the manuscript; **Qing Wang** and **Shuo Wang**: contributed to conducted experiments and data acquisition, analysis; **An Song** and **Yuan‐Yuan Wang**: contributed to data acquisition, analysis and revised the manuscript; **Hao Li** and **Zhi‐Jun Sun**: contributed to conception, design, data analysis, and drafted and revised the manuscript. All authors provided final approval and agreed to be responsible for all aspects of the study.

## FUNDING INFORMATION

This study was financial supported by the National Natural Science Foundation of China 82303328 (H.L.), 82273202 (Z.J.S.), and 82072996 (Z.J.S.), the Fundamental Research Funds for the Central Universities 2042023kf0141 (H.L.), 2042022dx0003 (Z.J.S.), and 2042024kf0021 (Z.J.S.), National Key Research and Development Program 2022YFC2504200 (Z.J.S.), Young Elite Scientist Support Program by CSA 2023PYRC001 (H.L.) and Natural Science Foundation of Wuhan 2023020201020516 (H.L.).

## CONFLICT OF INTEREST STATEMENT

The authors have declared that no conflict of interest exists.

## Supporting information


Data S1.

## Data Availability

The data supporting the present study are available from the corresponding author upon reasonable request.
