# Supplementary material for: Oncolytic herpes simplex virus propagates tertiary lymphoid structure formation via CXCL10/CXCR3 to boost antitumor immunity
Source: Cell Prolif. 2024 Sep 1;58(1):e13740. doi: 10.1111/cpr.13740 (PMC11693575; doi:10.1111/cpr.13740)
Supplement: Supplementary file 1 — Data S1. [file CPR-58-e13740-s001.docx]

**Supplemental information**

**Oncolytic herpes simplex virus induces tertiary lymphoid structure formation via CXCL10/CXCR3 to boost antitumor immunity**

Meng-Jie Zhang^1,#^, Wen-Ping Lin^1,#^, Qing Wang^1^, Shuo Wang^1^, An Song^1^, Yuan-Yuan Wang^1^, Hao Li^1,2,*^, Zhi-Jun Sun^1,2,*^

^1^ State Key Laboratory of Oral & Maxillofacial Reconstruction and Regeneration, Key Laboratory of Oral Biomedicine Ministry of Education, Hubei Key Laboratory of Stomatology, School & Hospital of Stomatology, Frontier Science Center for Immunology and Metabolism, Taikang Center for Life and Medical Sciences, Wuhan University, Wuhan, China.

^2^ Department of Oral Maxillofacial-Head Neck Oncology, School & Hospital of Stomatology, Wuhan University, Wuhan, China.

^#^ Authors contributing equally to this article.

**Supplementary Materials and Methods**

**Quantitative RT-PCR (qRT-PCR)**

The total RNA was isolated from the tumor tissues of mice according to the protocol provided by Axygen. The RNA concentration was measured by a spectrometer (Shimadzu UV-2401PC). The total RNA was isolated from the tumor tissues and the RNA concentration was measured. According to the manufacturer's instructions, 1 μg of total RNA was reverse transcribed using HiScript II Reverse Transcriptase (Vazyme Biotech). Subsequently, 1% of the cDNA was used as template in each RT-PCR with SYBR master mix (Vazyme Biotech). The amplification protocol consisted of an initial denaturation at 95 °C for 3 min, followed by 40 cycles of 95 °C for 10 s and 60 °C for 30 s, with a final extension consisting of 95 °C for 15 s, 60 °C for 1 min, and an indefinite hold at 4 °C. The relative expression levels of *Ccl2, Ccl3, Ccl4, Ccl5, Ccl8, Ccl19, Cxcl9, Cxcl10, Cxcl11, Cxcl13, Cxcr3* and *Ccl21* were quantified and normalized against β-actin as a reference gene. The sequences of primers used are provided Supplemental Table 1.

**Analysis of infiltrating immune cells and gene expression.**

For the assessment of CXCL10 and CXCR3 expression and their correlation with the TME, we collected the pertinent clinical characteristics of HNSCC patients from TCGA (https://portal.gdc.cancer.gov/). Subsequently, we calculated the enrichment scores for the TCGA-HNSCC database and applied them to perform CIBERSORT (http://cibersort.stanford.edu/).

**Figure S1**


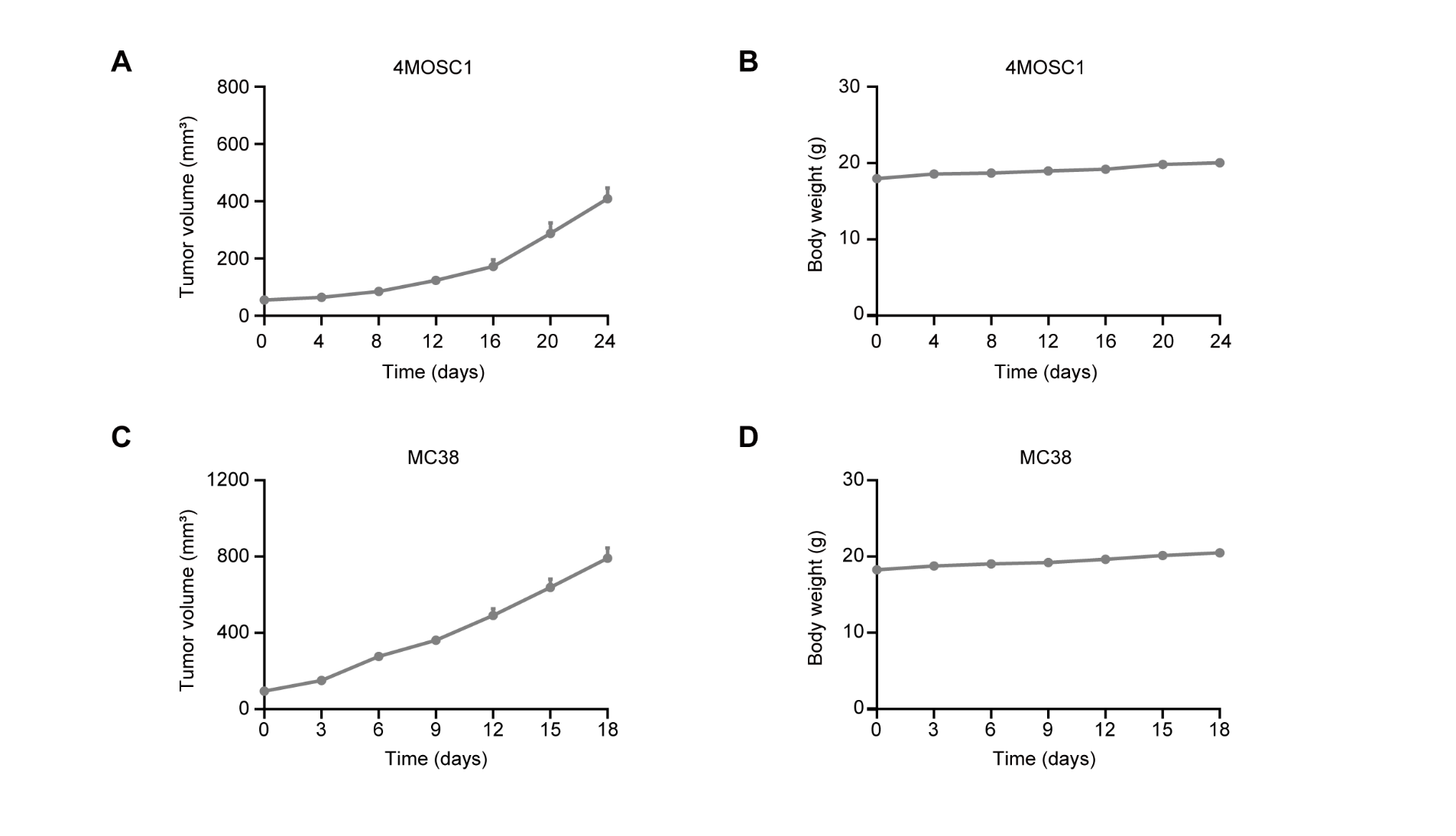


**Figure S1: (A)** Recording the average tumor growth curves of 4MOSC1 tumor-bearing mouse (n = 16). **(B)** Recording the average body weight curve of 4MOSC1tumor-bearing mouse (n = 16). **(C)** Recording the average tumor growth curves of MC38 tumor-bearing mouse (n = 16). **(D)** Recording the average body weight curve of MC38 tumor-bearing mouse (n = 16).

**Figure S2**


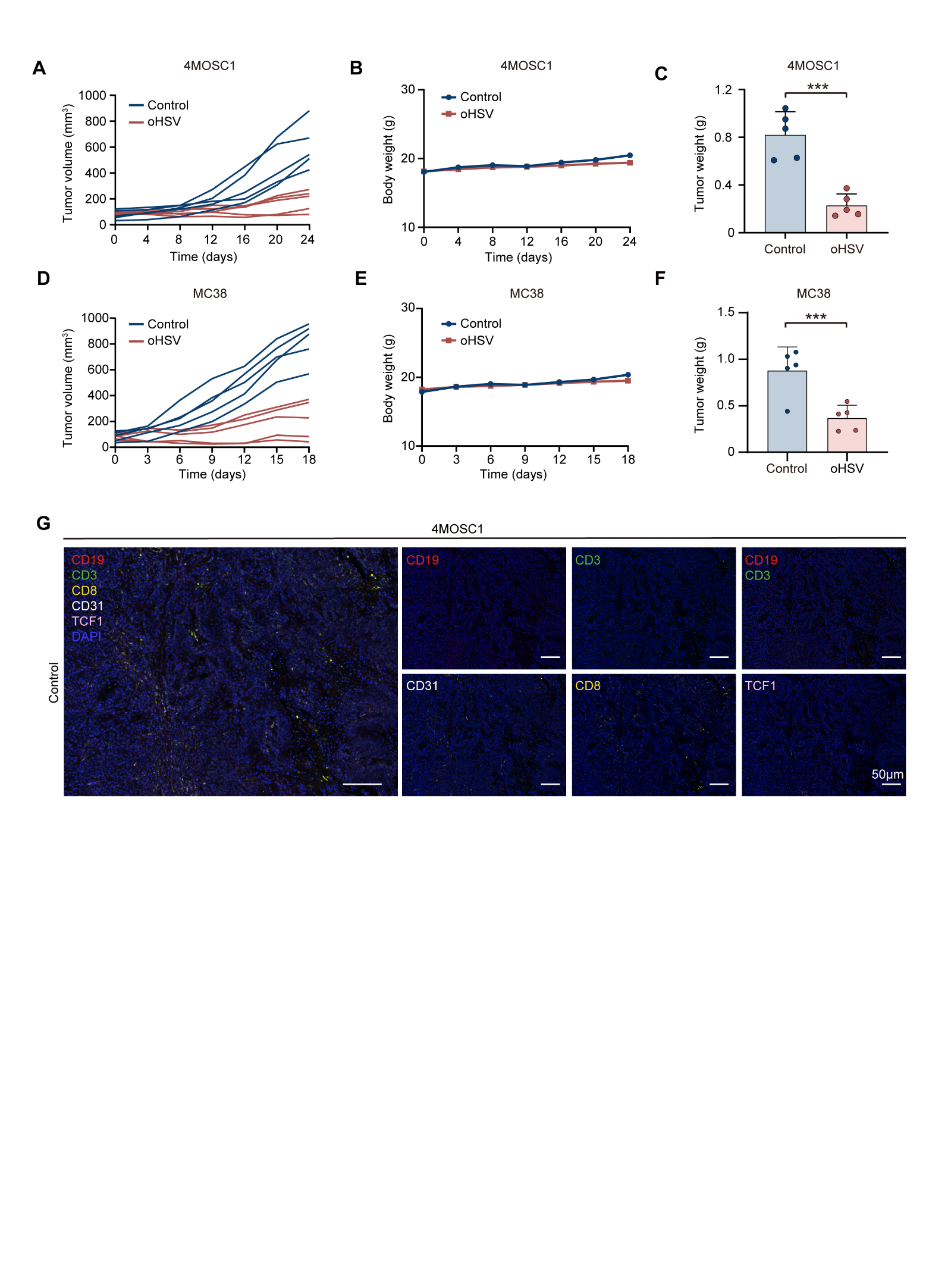


**Figure S2: (A)** Recording the tumor growth curves of 4MOSC1 tumor-bearing mouse in different groups (n = 5, each group). **(B)** Recording the average body weight curves of 4MOSC1 tumor-bearing mouse in different groups. **(C)** Recording the average tumor weights of 4MOSC1 tumor-bearing mouse in different groups. **(D)** Recording the tumor growth curves of MC38 tumor-bearing mouse in different groups (n = 5, each group). **(E)** Recording the average body weight curves of MC38 tumor-bearing mouse in different groups. **(F)** Recording the average tumor weights of MC38 tumor-bearing mouse in different groups. **(G)** Representative mIHC images of CD19 (red), CD3 (green), CD8 (yellow), CD31 (white), TCF1 (pink) in 4MOSC1-bearing mouse of control group. Statistical analysis is used by two-tailed unpaired *t* test. The above data are presented as mean ± SD (****P* < 0.001). Scale bar, 50 μm.

**Figure S3**


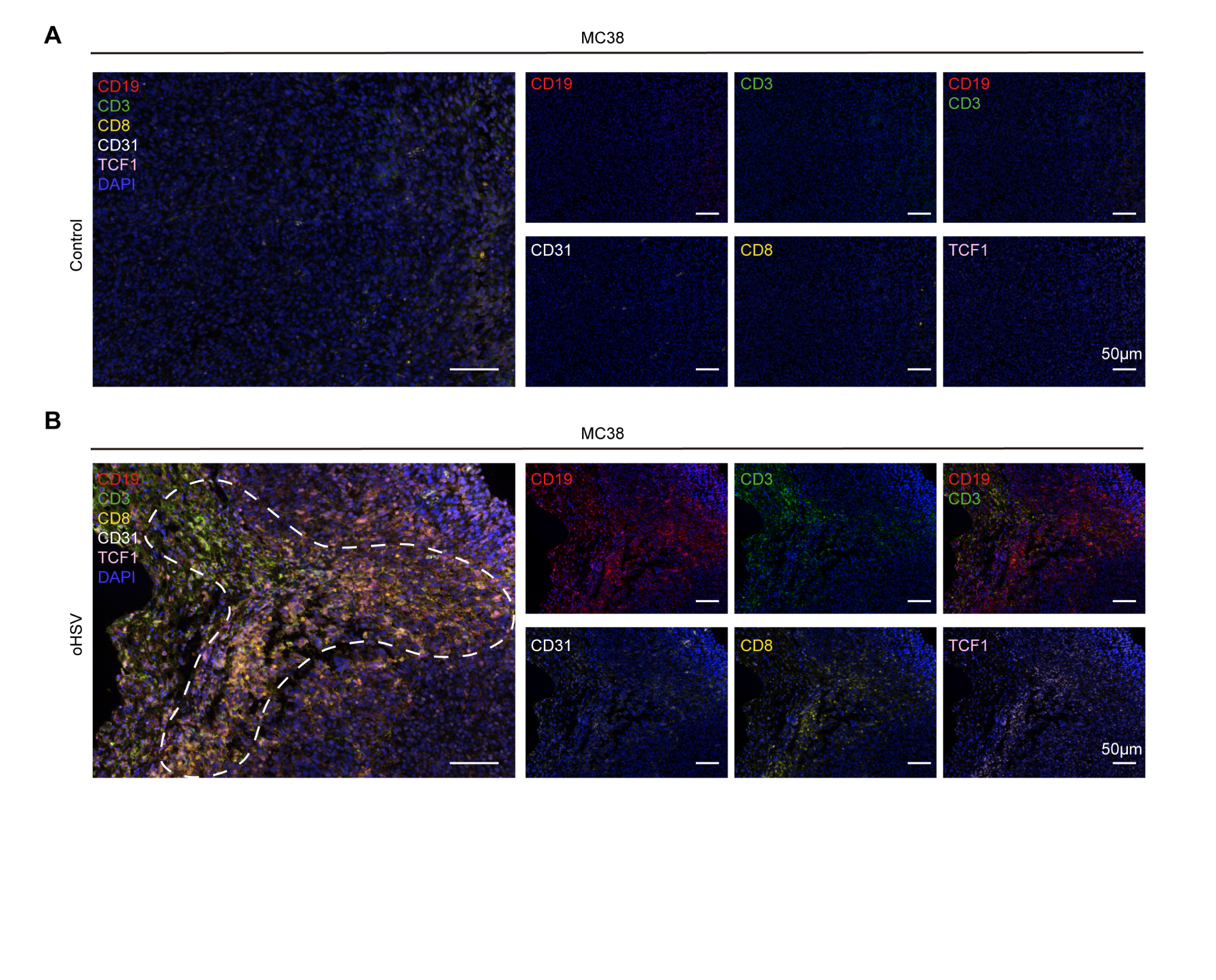


**Figure S3:** Representative mIHC images of CD19 (red), CD3 (green), CD8 (yellow), CD31 (white), TCF1 (pink) in MC38 tumor-bearing mouse of control group **(A)** and oHSV group **(B)**. Scale bar, 50 μm.

**Figure S4**


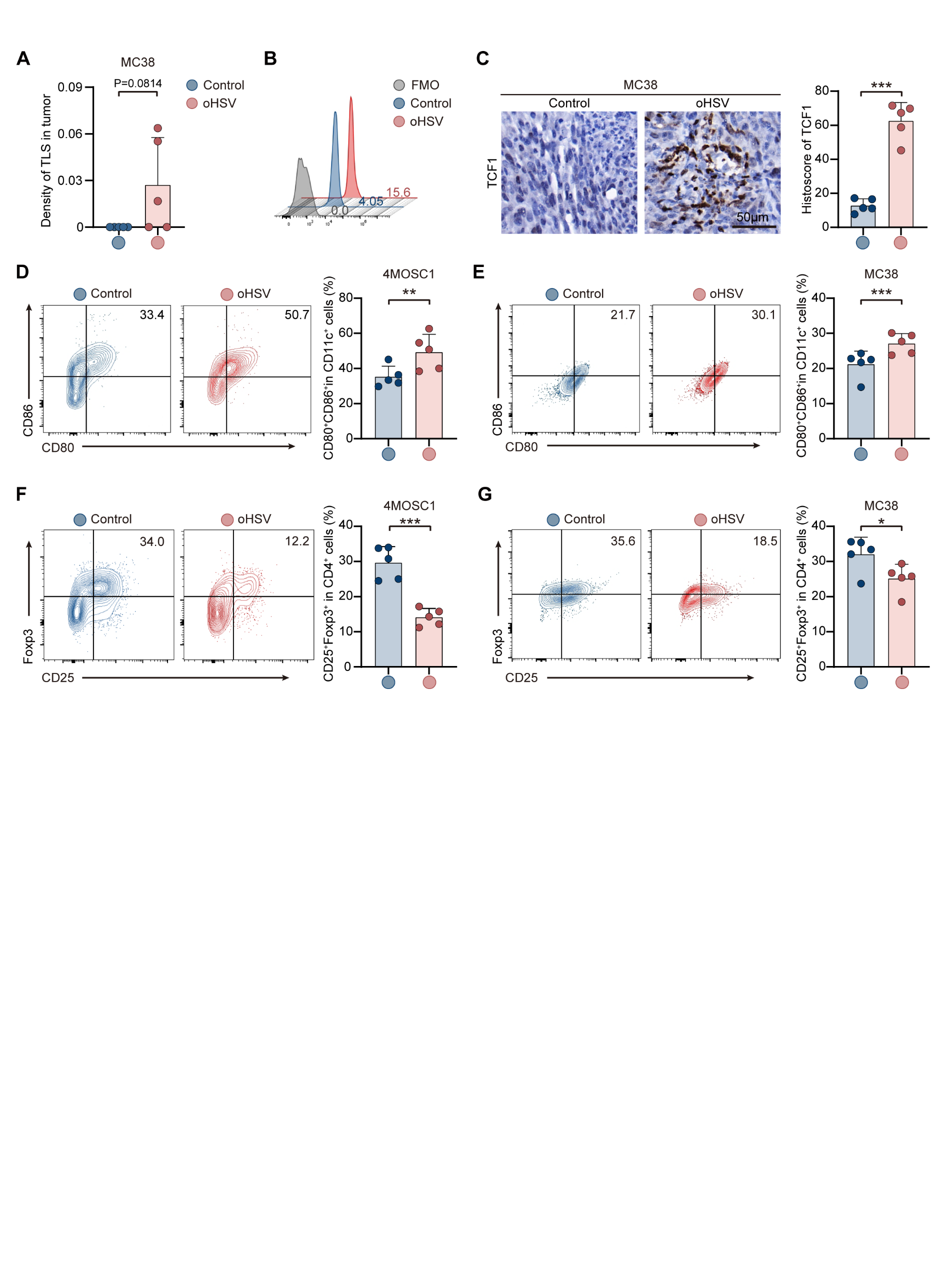


**Figure S4: (A)** Quantifying the density of TLS in control and oHSV groups of MC38 tumors. **(B)** Representative flow cytometry images of TCF-1^+^ in CD8^+^ cells in MC38 tumors. **(C)** Representative immunohistochemistry images and quantification of immunohistochemistry of TCF1 in MC38 tumor bearing mouse. **(D, E)** Analyzing images using flow cytometry and quantifying CD80^+^CD86^+^ in CD11c^+^ cells in 4MOSC1 and MC38 tumors. **(F, G)** Analyzing images using flow cytometry and quantifying CD25^+^Foxp3^+^ in CD4^+^ T cells in 4MOSC1 and MC38 tumors. Statistical analysis is used by two-tailed unpaired *t* test. The results are presented as mean ± SD (**P* < 0.05, ***P* < 0.01 and ****P* < 0.001). Scale bar, 50 μm.

**Figure S5**


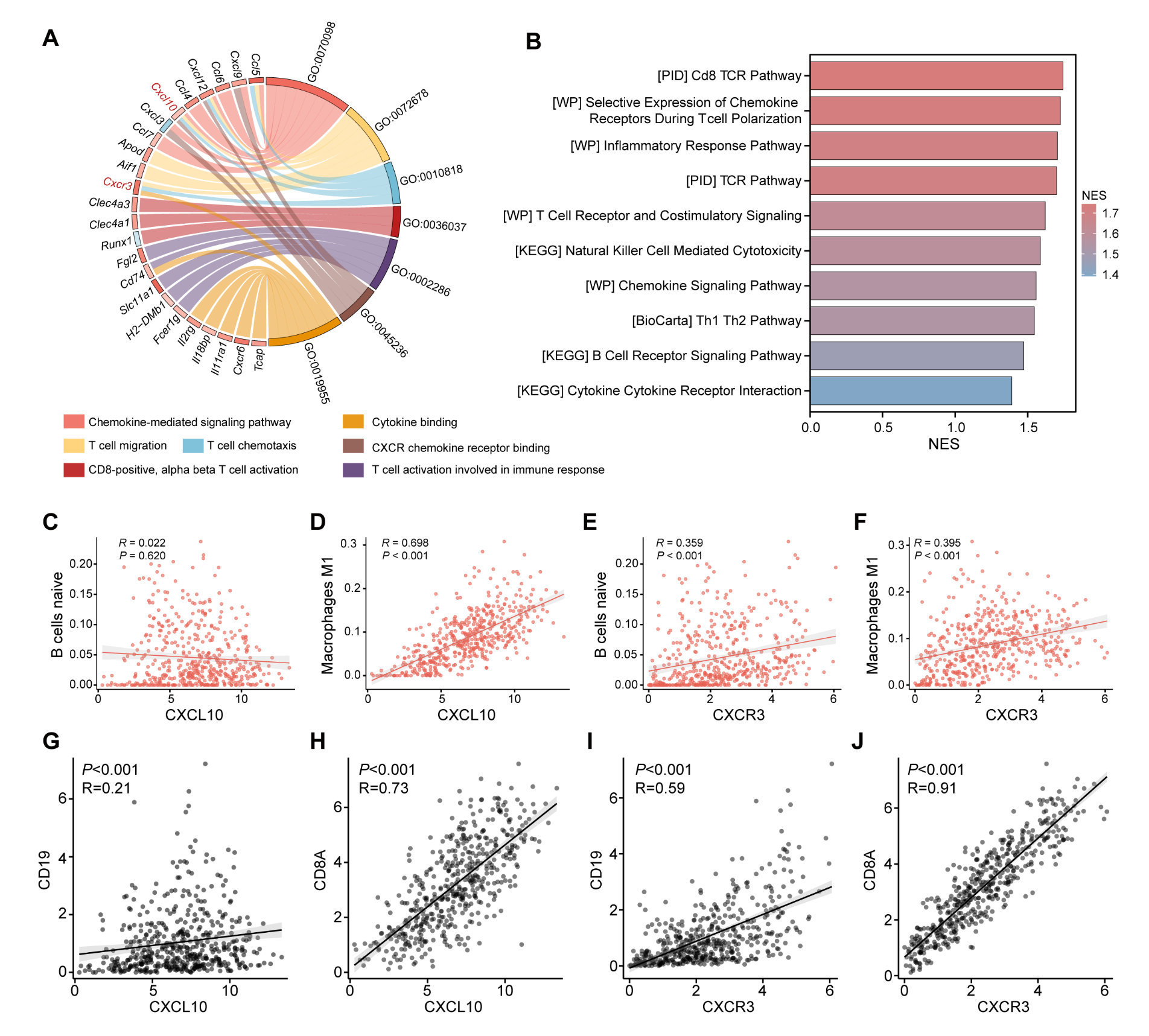


**Figure S5: (A)** GO term and KEGG pathway enrichment of Chord diagram. **(B)** The enriched score for 4MOSC1 tumors in control and oHSV group. **(C, D)** The correlation of CXCL10 gene expression with B cells and M1 macrophage. **(E, F)** The correlation of CXCR3 gene expression with B cells and M1 macrophage. **(G, H)** The correlation of CXCL10 expression with CD19, and CD8A in HNSCC-TCGA database. **(I, J)** The correlation of CXCR3 expression with CD19, and CD8A in HNSCC-TCGA database. Correlation was determined by Spearman’s correlation test.

**Figure S6**


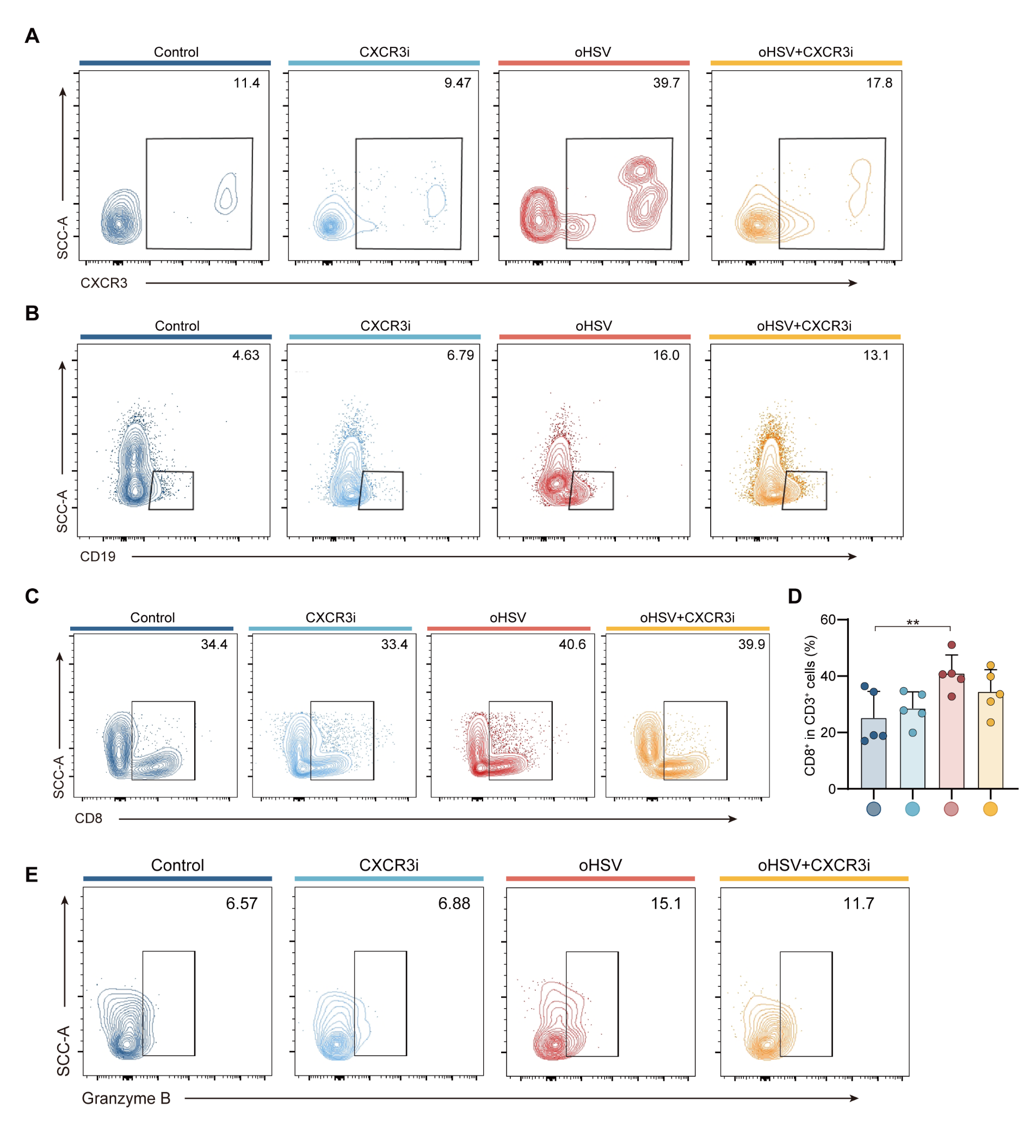


**Figure S6: (A)** Representative flow cytometry images of CXCR3^+^ in CD8^+^ T cells in 4MOSC1 tumors. **(B)** Representative flow cytometry images of CD19^+^ in CD45^+^ cells in 4MOSC1 tumors. **(C, D)** Analyzing images using flow cytometry and quantifying CD8^+^ in CD3^+^ cells in 4MOSC1 tumors. **(E)** Representative flow cytometry images of granzyme B^+^ in CD8^+^ cells in 4MOSC1 tumors. Statistical analysis is used by one-way ANOVA with Tukey’s multiple comparisons test. The results are presented as mean ± SD (***P* < 0.01).

**Figure S7**


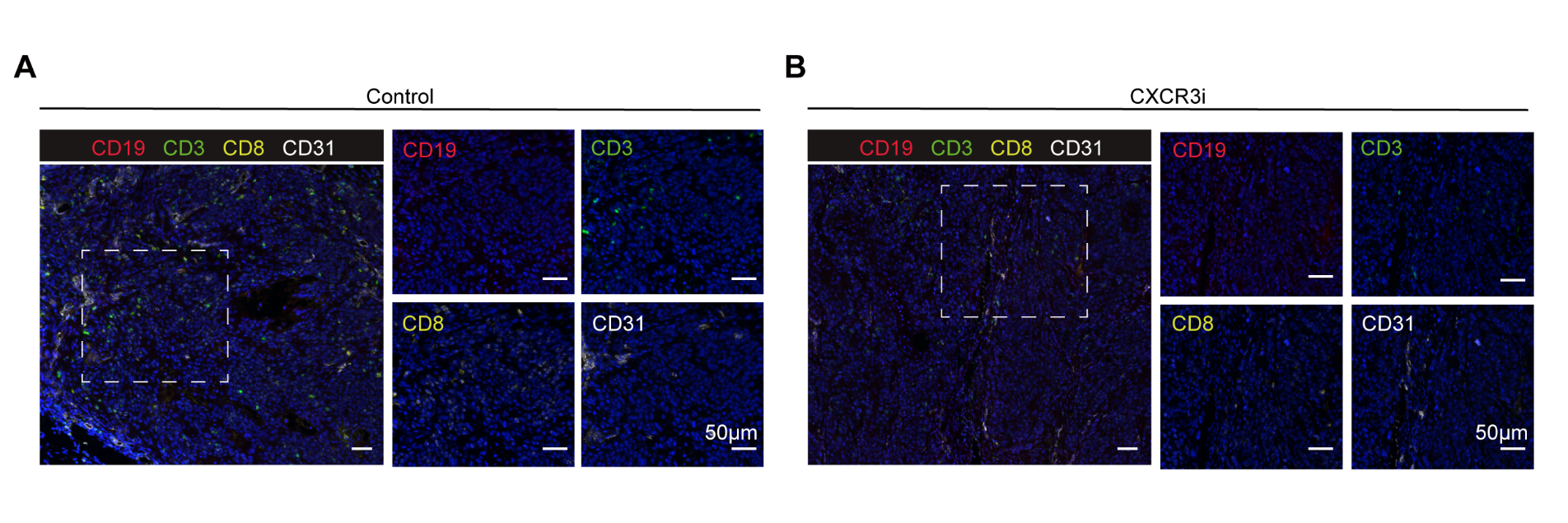


**Figure S7:** Representative mIHC images of CD19 (red), CD3 (green), CD8 (yellow), CD31 (white), in 4MOSC1 tumor-bearing mouse of control group **(A)** and CXCR3i group **(B)**. Scale bar, 50 μm.

**Figure S8**


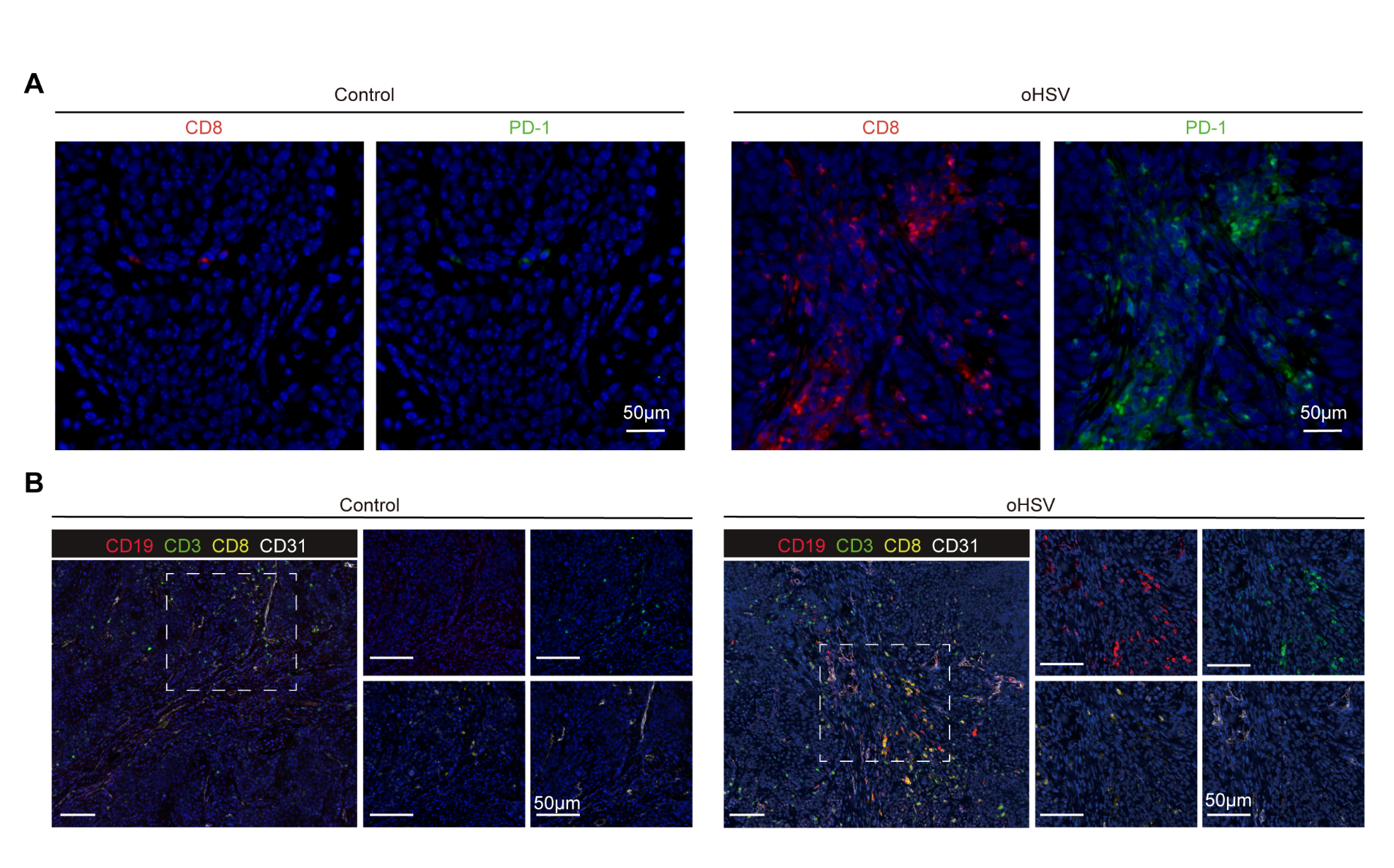


**Figure S8: (A)** Representative immunofluorescence images of CD8 (red) and PD-1 (green) in control and oHSV groups of 4MOSC1 tumors. **(B)** Representative mIHC images of CD19 (red), CD3 (green), CD8 (yellow), CD31 (white) in 4MOSC1 tumor-bearing mouse of control and oHSV group. Scale bar, 50 μm.

**Figure S9**


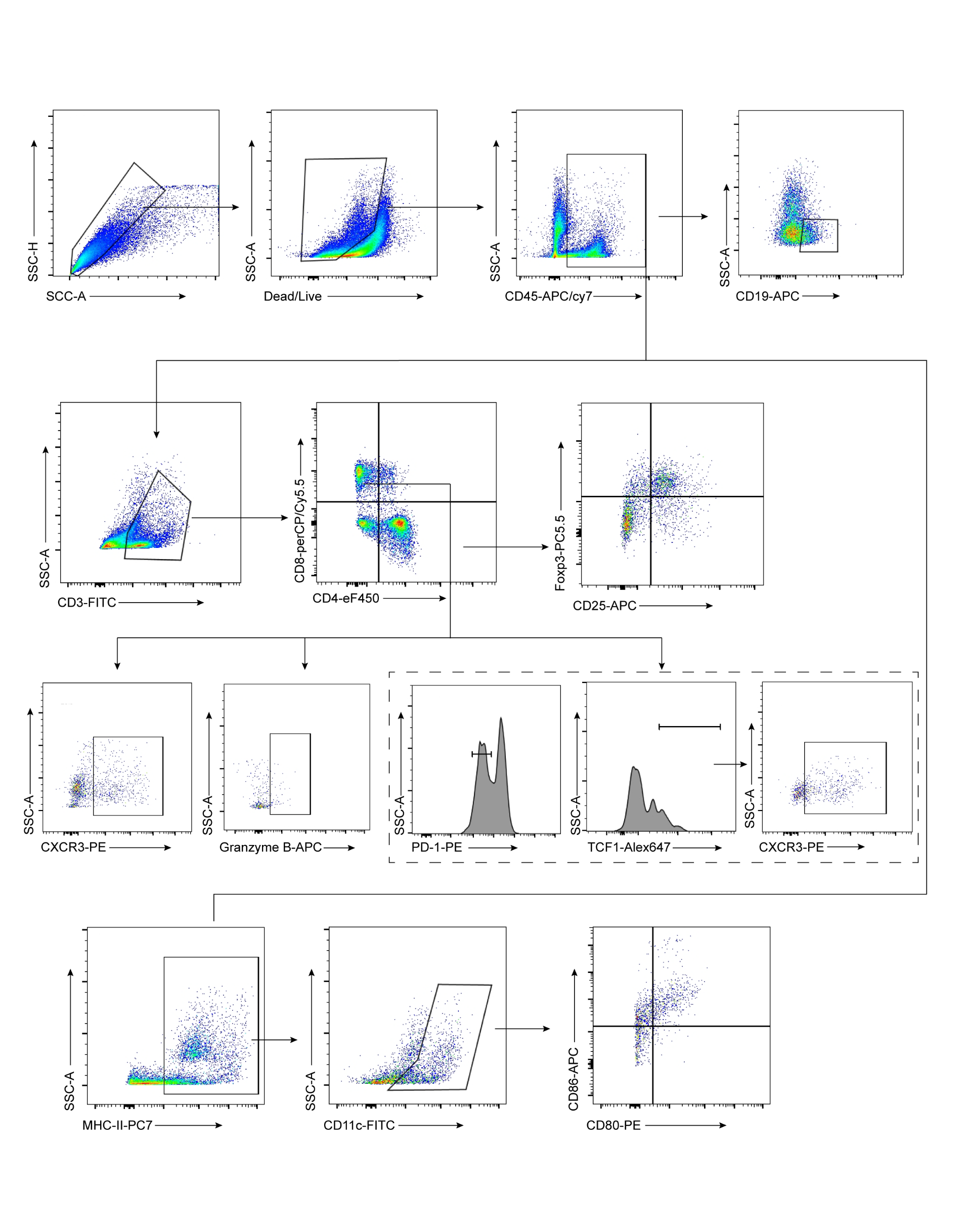


**Figure S9:** Gating strategy used to identify the CD45^+^CD19^+^ cells (B cells), CD45^+^CD8^+^ cells, CXCR3^+^CD3^+^CD8^+^ cells (T cells), CD25^+^Foxp3^+^CD3^+^CD4^+^ cells (Treg cells), TCF1^+^ CD8^+^ cells and PD-1^int^CD8^+^ cells (CD8^+^ Tpex), granzyme B^+^CD3^+^CD8^+^ cells, and CD80^+^CD86^+^ cells (dendritic cells) from tumor tissue.

**Table S1:** Sequence of primers used in this study.

| *Ccl2* | Forward | TTAAAAACCTGGATCGGAACCAA |
| --- | --- | --- |
|  | Reverse | GCATTAGCTTCAGATTTACGGGT |
| *Ccl3* | Forward | TTCTCTGTACCATGACACTCTGC |
|  | Reverse | CGTGGAATCTTCCGGCTGTAG |
| *Ccl4* | Forward | TTCCTGCTGTTTCTCTTACACCT |
|  | Reverse | CTGTCTGCCTCTTTTGGTCAG |
| *Ccl5* | Forward | GCTGCTTTGCCTACCTCTCC |
|  | Reverse | TCGAGTGACAAACACGACTGC |
| *Ccl8* | Forward | TCTACGCAGTGCTTCTTTGCC |
|  | Reverse | AAGGGGGATCTTCAGCTTTAGTA |
| *Ccl19* | Forward | GGGGTGCTAATGATGCGGAA |
|  | Reverse | CCTTAGTGTGGTGAACACAACA |
| *Ccl21* | Forward | GTGATGGAGGGGGTCAGGA |
|  | Reverse | GGGATGGGACAGCCTAAACT |
| *Cxcl9* | Forward | TCCTTTTGGGCATCATCTTCC |
|  | Reverse | TTTGTAGTGGATCGTGCCTCG |
| *Cxcl10* | Forward | CTCATCCTGCTGGGTCTGAG |
|  | Reverse | CCTATGGCCCTCATTCTCAC |
| *Cxcl11* | Forward | GGCTTCCTTATGTTCAAACAGGG |
|  | Reverse | GCCGTTACTCGGGTAAATTACA |
| *Cxcl13* | Forward | GGCCACGGTATTCTGGAAGC |
|  | Reverse | GGGCGTAACTTGAATCCGATCTA |
| *Cxcr3* | Forward | TACCTTGAGGTTAGTGAACGTCA |
|  | Reverse | CGCTCTCGTTTTCCCCATAATC |
| *β*-Actin | Forward | TCTACGAGGGCTATGCTCTCC |
|  | Reverse | TCTTTGATGTCACGCACGATTTC |

**Table S2:** GESA enrichment analysis associated with chemokines and immune activation.

| ID | Set Size | Enrichment Score | NES | *P* value | *P. adjust* | Q value |
| --- | --- | --- | --- | --- | --- | --- |
| KEGG Chemokine Signaling Pathway | 174 | 0.5574 | 1.4979 | 0.0009 | 0.0222 | 0.0198 |
| REACTOME PD-1 Signaling | 20 | 0.8042 | 1.6770 | 0.0007 | 0.0182 | 0.0162 |
| PID CD8 TCR Pathway | 54 | 0.7186 | 1.7441 | 3.09e-05 | 0.0030 | 0.0026 |
| WP Chemokine Signaling Pathway | 165 | 0.5811 | 1.5577 | 0.0001 | 0.0074 | 0.0066 |
| PID TCR Pathway | 63 | 0.6855 | 1.6993 | 0.0002 | 0.0084 | 0.0075 |
| KEGG Natural Killer Cell Mediated Cytotoxicity | 119 | 0.6105645 | 1.5878 | 0.0002 | 0.0084 | 0.0075 |
| WP Selective Expression of Chemokine Receptors During T cell Polarization | 29 | 0.7713 | 1.7255 | 0.0003 | 0.0123 | 0.0109 |
| WP Inflammatory Response Pathway | 30 | 0.7603 | 1.7048 | 0.0005 | 0.0175 | 0.0156 |
| KEGG Cytokine Receptor Interaction | 241 | 0.5093 | 1.3907 | 0.0034 | 0.0489 | 0.0436 |
| WP T cell Receptor and Costimulatory Signaling | 28 | 0.7281 | 1.6209 | 0.0042 | 0.0582 | 0.0518 |
| KEGG B Cell Receptor Signaling Pathway | 75 | 0.5844 | 1.4729 | 0.0048 | 0.0660 | 0.0588 |
| BioCarta Th1Th2 Pathway | 19 | 0.7479 | 1.5458 | 0.0083 | 0.0931 | 0.0829 |

**Table S3:** The enriched mouse GO terms for oHSV group associated with immune activation.

| Ontology | ID | Description | Gene Ratio | Bg Ratio | *P* value | *P. adjust* |
| --- | --- | --- | --- | --- | --- | --- |
| BP | GO:0002253 | Activation of immune response | 24/246 | 446/28814 | 1.01e-12 | 2.24e-10 |
| BP | GO:0050863 | Regulation of T cell activation | 18/246 | 348/28814 | 1.43e-09 | 1.09e-07 |
| BP | GO:0002468 | Dendritic cell antigen processing and presentation | 6/246 | 17/28814 | 4.17e-09 | 2.94e-07 |
| BP | GO:0070098 | Chemokine-mediated signaling pathway | 8/246 | 62/28814 | 5.72e-08 | 2.72e-06 |
| BP | GO:0050729 | Positive regulation of inflammatory response | 10/246 | 143/28814 | 4.53e-07 | 1.64e-05 |
| BP | GO:0042113 | B cell activation | 15/246 | 437/28814 | 6.09e-06 | 0.0001 |
| BP | GO:0036037 | CD8-positive, alpha-beta T cell activation | 3/246 | 34/28814 | 0.0030 | 0.0235 |
| BP | GO:0035710 | CD4-positive, alpha-beta T cell activation | 5/246 | 116/28814 | 0.0032 | 0.0243 |
| BP | GO:0009615 | Response to virus | 9/246 | 337/28814 | 0.0026 | 0.0208 |

**Table S4:** The enriched mouse GO terms combined with KEGG pathway associated with chemokines and immune activation.

| Ontology | ID | Description | Gene Ratio | Bg Ratio | *P* value | *P*. *adjust* | Z score |
| --- | --- | --- | --- | --- | --- | --- | --- |
| BP | GO:0070098 | Chemokine-mediated signaling pathway | 8/247 | 62/28814 | 5.9e-08 | 2.81e-06 | 2.1213 |
| BP | GO:0072678 | T cell migration | 6/247 | 63/28814 | 1.69e-05 | 0.0004 | 2.4495 |
| BP | GO:0010818 | T cell chemotaxis | 4/247 | 23/28814 | 4.11e-05 | 0.0007 | 2.0000 |
| BP | GO:0036037 | CD8-positive, alpha-beta T cell activation | 3/247 | 34/28814 | 0.0031 | 0.0234 | 0.5774 |
| BP | GO:0002286 | T cell activation involved in immune response | 5/247 | 124/28814 | 0.0044 | 0.0299 | 2.2361 |
| MF | GO:0045236 | CXCR chemokine receptor binding | 4/238 | 15/28275 | 6.21e-06 | 0.0002 | 1.0000 |
| MF | GO:0019955 | Cytokine binding | 7/238 | 143/28275 | 0.0002 | 0.0035 | 2.6458 |
